# Multimorbidity phenotypes and associated characteristics in severe asthma: an observational study of European severe asthma registries

**DOI:** 10.1016/j.lanepe.2026.101600

**Published:** 2026-02-05

**Authors:** Anna Freeman, Saša Rink, Aruna T. Bansal, Betty Frankemölle, Mehar Singh, Jacob K. Sont, Apostolos Bossios, Ben Ainsworth, Michael Hyland, Rekha Chaudhuri, Dace Matisa, Florin Mihaltan, Antonio Spanevello, Enrico Heffler, Ian Adcock, Martina Zappa, Giorgio Walter Canonica, Guy Brusselle, Arnaud Bourdin, Giulia Anna Maria Luigia Costanzo, Ildiko Horvath, Dóra Lúðvíksdóttir, Stefania Principe, Peter Kopač, Cláudia Chaves Loureiro, Salman Siddiqui, Arne Egesten, Virginija Kalinauskaite-Zukauske, Sanja Dimic-Janjic, Graham Roberts, Sanja Hromis, Branislava Milenkovic, Judit Varkonyi-Sepp, Ozlem Goksel, Ana M. Pereira, Ratko Djukanovic, Angela Rizzi, Marco Caminati, Ruihua Hou, Anamarija Štajduhar, Dóra Paróczai, Luisa Brussino, Liam Heaney, Hans Michael Haitchi, Matteo Bonini, Kristina Bieksiene, Ebru Damadoglu, Valentyna Yasinska, Bilun Gemicioglu, Sanja Popović Grle, Anneke ten Brinke, Zsuzsanna Csoma, Iveta Kroica, Piotr Kuna, Barbro Dahlen, Celeste Porsbjerg, Hilary Hodge, Sabina Škrgat, Florence Schleich, Ramesh J. Kurukulaaratchy

**Affiliations:** a School of Clinical and Experimental Sciences, Faculty of Medicine, University of Southampton, UK; b National Institute for Health Research (NIHR) Southampton Biomedical Research Centre at University Hospital Southampton NHS Foundation Trust, Southampton, UK; c Respiratory Medicine Department, University Hospital Southampton NHS Foundation Trust, Southampton, UK; d Department of Pulmonary Diseases and Allergy, University Medical Centre Ljubljana, Slovenia; e Acclarogen Ltd, St John's Innovation Centre, Cambridge, UK; f European Lung Foundation, UK; g Dept of Biomedical Data Sciences, Leiden University Medical Centre, Leiden, the Netherlands; h Karolinska Severe Asthma Centre, Department of Respiratory Medicine and Allergy, Karolinska University Hospital, Stockholm, Sweden; i Division for Lung and Airway Research, Institute for Environmental Medicine, Karolinska Institutet, Stockholm, Sweden; j Lung Laboratory, Centre for Molecular Medicine, Karolinska University Hospital, Stockholm, Sweden; k School of Psychology, Faculty of Environmental and Life Sciences, University of Southampton, Southampton, UK; l School of Psychology, University of Plymouth, Plymouth, UK; m School of Infection and Immunity, University of Glasgow, Scotland; n Riga East University Hospital, Latvia; o Național Institute of Pneumology, Bucharest, Romania; p Department of Cardiopulmonary Rehabilitation, Istituti Clinici Scientifici Maugeri IRCCS, Tradate, Italy; q Department of Medicine and Surgery, University of Insubria, Varese, Italy; r Personalized Medicine, Asthma and Allergy - IRCCS Humanitas Research Hospital, Rozzano, MI, Italy; s Department of Biomedical Sciences, Humanitas University, Pieve Emanuele, MI, Italy; t National Heart & Lung Institute, Imperial College London, UK; u Department of Respiratory Medicine, Ghent University Hospital, Ghent, Belgium; v Department of Respiratory Diseases, Montpellier University Hospital, Arnaud de Villeneuve Hospital, Montpellier, France; w PhyMedExp (INSERM U 1046, CNRS UMR9214), Montpellier University, Montpellier, France; x Department of Medical Science and Public Health, University of Cagliari, Cagliari, Italy; y National Koranyi Institute of Pulmonology, Budapest, Hungary; z Department of Respiratory Medicine, Landspitali University Hospital and University of Iceland, Iceland; aa Amsterdam University Medical Centre, the Netherlands; ab University Clinic of Respiratory Diseases Golnik, Slovenia; ac Faculty of Medicine, University of Ljubljana, Slovenia; ad Pneumology Unit, Hospitais da Universidade de Coimbra, Centro Hospitalar e Universitário de Coimbra, Coimbra, Portugal; ae Department of Clinical Sciences Lund, Respiratory Medicine, Allergology, & Palliative Medicine, Lund University and Skåne University Hospital, Lund, Sweden; af Department of Pulmonology, Lithuanian University of Health Sciences, Kaunas, Lithuania; ag Faculty of Medicine, University of Belgrade, Belgrade, Serbia; ah Clinic for Pulmonary Diseases, University Clinical Centre of Serbia, Belgrade, Serbia; ai The David Hide Asthma & Allergy Research Centre, St Mary's Hospital, Newport, Isle of Wight, UK; aj Institute for Pulmonary Diseases of Vojvodina, Sremska Kamenica, Serbia; ak Faculty of Medicine, University in Novi Sad, Serbia; al Clinical Health Psychology Department, Southern Health NHS Foundation Trust/University Hospital Southampton NHS Foundation Trust, Southampton, UK; am Department of Pulmonary Diseases, Ege University Faculty of Medicine, İzmir, Türkiye; an MEDCIDS-Department of Community Medicine, Information and Health Decision Sciences, Faculty of Medicine, University of Porto, Porto, Portugal; ao PaCeIT – Patient Centred Innovation and Technologies, CINTESIS@RISE - Health Research Network, Faculty of Medicine, University of Porto, Porto, Portugal; ap Allergy Unit, Instituto and Hospital CUF, Porto, Portugal; aq UOSD Allergologia e Immunologia Clinica. Dipartimento Scienze Mediche e Chirurgiche. Fondazione Policlinico Universitario Agostino Gemelli IRCCS, Rome, Italy; ar Department of Medicine, University of Verona, Verona, Italy; as Clinic for pulmonary diseases Jordanovac, University Hospital Centre Zagreb, Croatia; at University of Zagreb School of Medicine, as an external collaborator; au Department of Pulmonology, University of Szeged, Hungary; av Allergy and Immunology unit, Department of medical sciences, University of Turin, Italy; aw Wellcome-Wolfson Centre for Experimental Medicine, Queen's University Belfast, UK; ax Department of Public Health and Infectious Diseases, Sapienza University of Rome, Italy; ay Department of Pulmonology, Division of Allergy and Clinical Immunology, Hacettepe University, School of Medicine, Ankara, Türkiye; az Clinical Lung and Allergy Research, Department of Medicine Huddinge, Karolinska Institutet, Stockholm, Sweden; ba Department of Pulmonary Diseases, Istanbul University-Cerrahpaşa, Cerrahpaşa Faculty of Medicine, Istanbul, Türkiye; bb Institute of Pulmonology and Tuberculosis, Istanbul University-Cerrahpaşa, Istanbul; bc School of Medicine, University of Zagreb, Zagreb, Croatia; bd Department of Respiratory Medicine, Medical Centre Leeuwarden, Leeuwarden, the Netherlands; be Medical University of Lodz, Poland; bf Department of Respiratory Medicine, Karolinska University Hospital, Stockholm, Sweden; bg Lab of Exercise Physiology, University of Liege, Belgium; bh Department of Respiratory and Infectious Diseases, Bispebjerg Hospital, Copenhagen, Denmark; bi Institute of Clinical Medicine, University of Copenhagen, Copenhagen, Denmark

**Keywords:** Severe asthma, Multimorbidity, Cluster, Phenotype

## Abstract

**Background:**

The phenotypic nature of multimorbidity in severe asthma is poorly understood. Our aims in this study were to define multimorbidity phenotypes and their characteristics in severe asthma across Europe by identifying and characterising co-aggregation of comorbidities.

**Methods:**

Cross-sectional patient data were analysed from the pan-European Severe Heterogenous Asthma Research Collaboration: Patient Centred (SHARP) Central database of national severe asthma registries. Patients were grouped by four European regions (North, South, East, and West). Hierarchical clustering of comorbidities was applied to characterise the correlation structure of the ten commonest comorbidities within these geographical regions. Subsequent multimorbidity phenotypes (MMP) and their clinical features were then defined.

**Findings:**

Data were available for 2690 severe asthma patients and 23 comorbidities from 11 countries. Three comorbidity clusters were consistently seen across the four European regions: 1) osteoporosis plus steroid-induced weight gain, 2) eczema plus rhinitis, and 3) chronic sinusitis plus nasal polyps. Four further comorbidities (obesity, bronchiectasis, gastro-oesophageal reflux disease, psychological factors) showed variable clustering. Multimorbidity was ubiquitous. Patients were assigned multimorbidity phenotypes (MMP) according to comorbidity cluster alignment. MMP sn (sinonasal-associated) and MMP u (no specific cluster alignment) were commonest. MMP ster (steroid-associated multimorbidity) had highest maintenance oral steroid (m-OCS) use, and Body Mass Index, plus worst lung function, asthma control, and asthma exacerbation frequency. MMP max (maximal multimorbidity) showed high prevalence of variably assigned comorbidities, higher m-OCS and biologic treatment needs.

**Interpretation:**

Multimorbidity is common in severe asthma and can be classified into replicable novel phenotypes with characteristic clinical traits and outcomes. Recognising these phenotypes can guide better care of the ‘whole patient’ with severe asthma. Future clinical guidance should promote such understanding in order to support delivery of more effective personalised asthma care.

**Funding:**

10.13039/100008593European Respiratory Society, pharmaceutical industry partners (Sanofi, TEVA, Novartis, GlaxoSmithKline, Chiesi).

**Research in context**

**Evidence before this study**

Comorbidities are increasingly recognised as contributing to worse outcomes in difficult-to-treat/severe asthma and there is growing interest in the concept of multimorbidity as a means to understand these effects. However, there is limited understanding of the nature and impacts of multimorbidity in difficult-to-treat/severe asthma. We conducted a PubMed and Embase search on 1st January 2026 using the search terms ‘difficult-to-treat asthma’, ‘multimorbidity’, ‘comorbidity’ and ‘phenotype’, for studies published between 1st March 2015 and 1st January 2026. Numerous studies were identified that demonstrated the detrimental impacts of individual (more than 50 studies) and cumulative comorbidities (only four studies) on asthma outcomes. However, multimorbidity models for severe asthma have scarcely been studied. One recent publication presents a novel multimorbidity in difficult asthma score (MiDAS) that demonstrated associations between multimorbidity and worse asthma plus general health outcomes in difficult-to-treat asthma. MiDAS serves as a prompt for clinicians to recognise and address multimorbidity. However, we found no studies that offer clinicians a deeper understanding of the patterns or phenotypes of multimorbidity in difficult-to-treat/severe asthma and their clinical relevance.

**Added Value of this study**

To the best of our knowledge, we present the first study to define multimorbidity phenotypes in patients with severe asthma. This provides an evidence base to support a more holistic understanding of severe asthma. Using hierarchical cluster analysis, we identified three stable comorbidity clusters that were replicated across four geographical regions in Europe. These three clusters give rise to a multimorbidity phenotyping scheme in severe asthma. Patients allocated to these multimorbidity phenotypes demonstrated clinically distinct and relevant asthma-related clinical traits and clinical outcomes. Notable adverse outcome-associated multimorbidity phenotypes included the respective “unclustered”, “steroid-associated”, and “maximal” multimorbidity phenotypes. These phenotypes can be readily detected in clinical practice and their recognition could guide more holistic treatment approaches in severe asthma.

**Implications of all the available evidence**

Multimorbidity is commonplace, highly detrimental, and needs to be considered as a central component of difficult-to-treat/severe asthma management as highlighted by the recently published Multimorbidity in Difficult Asthma Score (MiDAS). The definition of distinct multimorbidity severe asthma phenotypes in the present study takes this understanding to the next level. Identification of multimorbidity phenotypes allows greater understanding of the nuances of multimorbidity in severe asthma which can promote personalised-medicine approaches to treat the ‘whole patient’. Taken collectively, this emerging evidence base provides clinicians with a useable framework to recognise and address patterns of multimorbidity in severe asthma management with the ultimate goal of improving future severe asthma care and patient outcome. Avoidance of over-exposure to oral corticosteroids alongside early consideration for biologic strategies in some patients, and/or implementation of multimodal approaches to address specific patterns of multimorbidity in others, emerge as critical clinical practice points in that regard.

## Introduction

The concept of multimorbidity, describing the co-existence of two or more comorbidities and their combined effects, is attracting increasing attention for long term health conditions.^1^ However, understanding of multimorbidity in relation to severe asthma is in its infancy. Most work in that regard has focused on the identification and burden of individual comorbidities in severe asthma,^2^^,^^3^ plus associations of simple cumulative comorbidity burden with worse asthma outcomes.^2^ From that body of work, it is evident that a holistic ‘treatable traits’ approach to severe asthma management based on a comprehensive systematic assessment, which identifies and addresses comorbidities, can considerably improve asthma outcomes including oral corticosteroid dependency.^4^^,^^5^ Conversely, current management approaches for severe asthma often remain singularly focused on the index airway condition.^1^ Yet, comorbidities are emerging as a factor that may influence biologic treatment responses in patients with severe asthma,^6^ with several comorbidities showing association with failure to achieve biologic-induced asthma remission. Notably, current definitions for asthma remission or treatment response fail to consider comorbidities.^7^

The recently published Multimorbidity in Difficult Asthma Score (MiDAS) provides compelling evidence of the key components of multimorbidity in difficult-to-treat/severe asthma and of their detrimental association with patient outcomes.^8^ It also offers clinicians a simple tool with which to identify patients whose asthma outcomes are at greatest risk from their multimorbidity. However, it remains unclear whether different patterns or phenotypes of multimorbidity occur in more severe asthma and how those might correlate with individual patient outcomes.

Cross-sectional analysis from the Severe Heterogenous Asthma Research Collaboration: Patient Centred (SHARP) demonstrated variability across Europe in characteristics, lifestyle factors, lung function and treatment regimens prior to biologic initiation for severe asthma patients.^9^ In this SHARP study, we sought to use a cluster analysis-based approach to identify comorbidity clusters in severe asthma that could be replicated across different geographic regions of Europe. We then used these clusters to distinguish and characterise multimorbidity patient phenotypes in severe asthma. We hypothesised that it would be possible to characterise clinically relevant multimorbidity phenotypes in severe asthma which would have distinct clinical features and associated outcomes.

## Methods

### Study design and participants

Cross-sectional observational patient data were drawn in April 2023 from the SHARP Central database of harmonised data variables from 11 European national severe asthma registries, as previously described^10^ and converted to the Observational Medical Outcomes Partnership Common Data Model (OMOP CDM). Most European registries included patients who fulfilled the severe asthma criteria according to the joint ERS/ATS guidelines^11^ but in some cases national asthma guidelines were used or all patients who attended specialist asthma centres were qualified for inclusion. The scope of reporting spans demographics, anthropometrics, smoking history, clinical measurements, asthma control (either Asthma Control Test [ACT] or Asthma Control Questionnaire [ACQ]), lung function, biomarkers, comorbidities, and medication. Eligibility criteria, and the nature of data shared, including trait definitions, were determined by individual registries; missing data were not imputed. Comorbidity diagnoses in each country reflected local clinical diagnostic criteria as captured in their respective severe asthma registries. Ethics approvals for SHARP were obtained through existing collaboration agreements with the individual participating national registries in SHARP. Patient consent for data sharing was obtained at point of individual registry consent. The study adhered to the Strengthening the Reporting of Observational Studies in Epidemiology (STROBE) guidelines.

### Statistical analysis

Patient characteristics were summarised using mean (SD), median (IQR), or n (%) for symmetrical continuously distributed data, non-symmetrical continuously distributed data and count data, respectively. The country-specific prevalence of each comorbidity was given as n (%).

Patients were assigned to a European region using the United Nations geoscheme for Europe^12^ as follows: North (Lithuania (LT), Latvia (LV), Sweden (SE)); East (Hungary (HU), Poland (PL) Romania (RO)); South (Croatia (HR), Serbia (RS), Slovenia (SL), Türkiye (TR)); West (Netherlands (NL)) (Supplementary Table S1). Baseline characteristics differed by region (Supplementary Table S2), and we wished to establish a consensus scheme across the spectrum of severe asthma in Europe. Comorbidities with prevalence of at least 5% in all four regions of Europe, were selected for detailed study.

#### Development of comorbidity clusters and multimorbidity patient phenotypes

Hierarchical clustering of variables^13^ was applied to create a hierarchical representation of the correlation structure of the comorbidities. An agglomerative approach was adopted in which the aggregation criterion was the decrease in homogeneity of clusters being merged. The homogeneity of a cluster was the sum of the correlation ratio between the variables and the centre of the cluster. In practical terms, the presence/absence of each comorbidity was used as input, and cluster analysis was conducted four times, by geographic region with the aim of establishing a consensus from independent groups. The cut-point of the hierarchy (number of clusters) was determined by inspection of scree plots and application of the elbow method (Supplementary Figure S4). Results were displayed using dendrograms, and consistency of co-aggregation was displayed using a Sankey diagram. An item response analysis was applied to assess the relative importance of the comorbidities in each cluster (Supplementary Figure S5).

Patients were then allocated into multimorbidity phenotypes based on which clusters were represented in their individual comorbidity profile. Summary statistics for patient multimorbidity phenotypes were provided via mean (SD), median (IQR), or n (%), as appropriate. All analyses were conducted using R version 4⸱2⸱3 or later (R Core Team, 2023).^14^

### Role of funding source

The funders of this study had no role in study design, data collection, analysis, data interpretation, or writing of the report.

## Results

### Demographic data, clinical characteristics and geographical variation

**Fig. 1:**
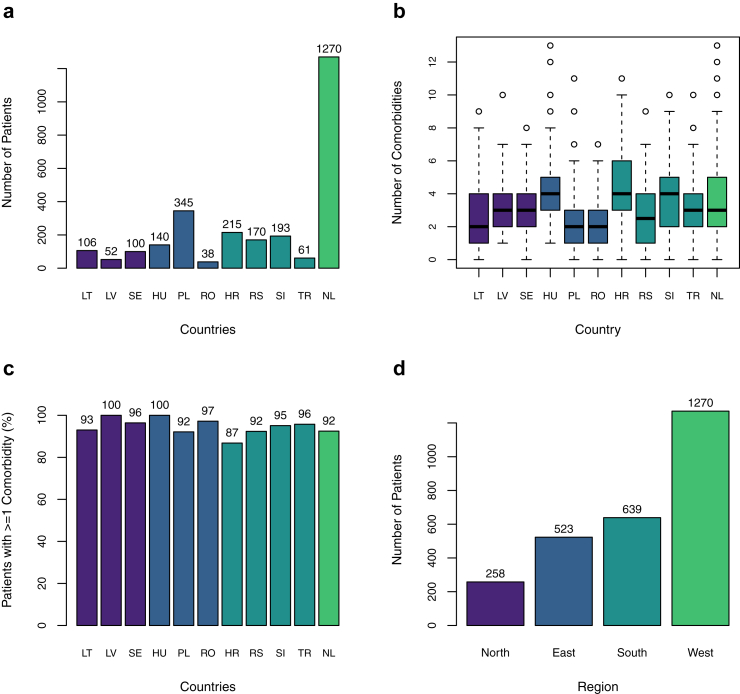
**Patient, comorbidity and multimorbidity counts by country plus regional patient distribution.** a) Patient counts by country; b) Boxplot of Number of Comorbidities per patient, by country; c) Percentage of patients with one or more comorbidity, by country; d) Number of patients by European region. Abbreviations HR; Croatia, HU; Hungary, LT Lithuania, LV; Latvia, NL; Netherlands, PL; Poland, RO; Romania, RS; Serbia, SE; Sweden, SI, Slovenia; TR; Türkiye.

Eleven countries contributed data for 2690 patients, of which 1270 were from the Netherlands, the oldest and largest severe asthma registry in this study (Fig. 1a).

The overall study population was predominantly female (60%), mean age 56-years, and 78% had adult-onset asthma. Disease severity was high with 70% reporting at least two exacerbations needing oral corticosteroids (OCS) in the past 12 months, 79% taking biologic therapy and 30% being on maintenance OCS (m-OCS). However patient demographics and clinical characteristics varied considerably across countries (Table 1 and Supplementary Results).

**Table 1:** Demographic data for participants by country as mean (SD) or median (interquartile range) or n (%).

Variable	HR N = 215	HU N = 140	LT N = 106	LV N = 52	NL N = 1270	PL N = 345	RO N = 38	RS N = 170	SE N = 100	SI N = 193	TR N = 61
Age at index date	58 (14)	56 (12)	58 (11)	60 (12)	56 (15)	55 (14)	51 (14)	53 (12)	59 (13)	57 (13)	49 (11)
Female	143 (67%)	94 (67%)	71 (67%)	37 (71%)	693 (55%)	227 (66%)	24 (63%)	117 (69%)	51 (51%)	109 (56%)	38 (62%)
Height (cm)	166 (12)	167 (11)	168 (10)	167 (10)	172 (11)	167 (11)	169 (6)	168 (9)	172 (11)	170 (10)	164 (10)
Weight (kg)	76 (17)	77 (16)	81 (16)	81 (23)	84 (17)	79 (19)	71 (13)	76 (17)	80 (18)	80 (18)	75 (13)
BMI (kg/m^2^)	27·3 (5·8)	27·4 (5·1)	29·0 (5·5)	29·0 (7·0)	28·1 (5·3)	28·6 (6·0)	24·8 (4·9)	26·7 (5·2)	26·9 (5·8)	27·8 (5·3)	27·8 (4·5)
Onset of Asthma											
Childhood onset	32 (15%)	32 (23%)	11 (12%)	9 (18%)	312 (27%)	78 (24%)	6 (17%)	26 (15%)	16 (17%)	25 (15%)	2 (3%)
Adult onset	181 (85%)	108 (77%)	84 (88%)	42 (82%)	836 (73%)	247 (76%)	29 (83%)	144 (85%)	80 (83%)	142 (85%)	59 (97%)
Smoking History											
Current smoker	18 (8%)	5 (4%)	6 (6%)	0 (0%)	14 (1%)	0 (0%)	2 (5%)	16 (9%)	0 (0%)	2 (1%)	1 (2%)
Previous smoker	66 (31%)	16 (11%)	20 (20%)	8 (16%)	537 (43%)	42 (12%)	10 (26%)	47 (28%)	20 (20%)	68 (37%)	10 (16%)
Never smoked	131 (61%)	119 (85%)	76 (75%)	43 (84%)	710 (56%)	302 (88%)	26 (68%)	107 (63%)	80 (80%)	113 (62%)	50 (82%)
Pack years	25 (12, 38)	10 (7, 12)	13 (5, 30)	15 (9, 24)	11 (5, 22)	10 (5, 20)	15 (5, 30)	15 (10, 30)	10 (5, 14)	15 (8, 28)	6 (5, 7)
Clinical Features											
Atopy	110 (51%)	75 (54%)	31 (29%)	13 (25%)	606 (48%)	142 (41%)	8 (21%)	98 (58%)	34 (34%)	67 (35%)	25 (41%)
FEV_1_ pre-BD as % predicted	63 (21)	64 (19)	68 (22)	67 (18)	80 (20)	65 (21)	67 (24)	69 (17)	76 (20)	72 (19)	72 (20)
FEV_1_ post-BD as % predicted	61 (20)	63 (15)	69 (22)	72 (20)	84 (20)	66 (21)	NA	70 (17)	74 (18)	69 (17)	73 (16)
FEV_1_/FVC as % predicted	73 (15)	72 (17)	74 (20)	73 (15)	82 (15)	71 (15)	NA	62 (11)	70 (15)	67 (17)	72 (13)
FeNO (ppb)	36 (20, 61)	34 (22, 53)	30 (21, 56)	22 (12, 39)	33 (19, 54)	32 (16, 50)	12 (4, 27)	38 (19, 71)	25 (18, 50)	49 (30, 79)	17 (16, 20)
Blood eosinophil count (x10^9^/L)	0·31 (0·1, 0·7)	0·50 (0·3, 0·8)	0·46 (0·2, 0·7)	0·52 (0·3, 0·7)	0·35 (0·2, 0·7)	0·23 (0·1, 0·5)	0·26 (0·1, 0·9)	0·41 (0·2, 0·7)	0·40 (0·1, 0·8)	0·36 (0·2, 0·6)	0·70 (0·4, 1·2)
Asthma control											
Well controlled	54 (34%)	44 (31%)	17 (17%)	5 (12%)	NA	45 (17%)	3 (27%)	30 (18%)	45 (49%)	80 (48%)	18 (36%)
Partly controlled	32 (20%)	58 (41%)	19 (19%)	6 (15%)	NA	73 (28%)	1 (9%)	47 (28%)	16 (18%)	28 (17%)	7 (14%)
Poorly controlled	73 (46%)	38 (27%)	62 (63%)	30 (73%)	NA	140 (54%)	7 (64%)	90 (54%)	30 (33%)	59 (35%)	25 (50%)
≥2 exacerbations/yr	115 (66%)	108 (77%)	86 (91%)	44 (86%)	799 (66%)	119 (46%)	13 (65%)	153 (91%)	41 (93%)	156 (82%)	50 (93%)
Biologic treatment	124 (58%)	70 (50%)	79 (75%)	27 (52%)	1148 (90%)	280 (81%)	23 (61%)	111 (65%)	82 (82%)	132 (68%)	46 (75%)
Maintenance OCS, n (%)	77 (36%)	23 (16%)	11 (10%)	15 (29%)	418 (33%)	48 (14%)	7 (18%)	67 (39%)	19 (19%)	91 (47%)	29 (48%)

**Abbreviations:** BMI; body mass index, cm; centimetre, FeNO, fractional exhaled nitric oxide, FEV_1_; forced expiratory volume in 1 s, FVC; forced vital capacity, HR; Croatia, HU; Hungary, LT; Lithuania, LV; Latvia, NL, Netherlands, OCS; oral corticosteroids, PL; Poland, PP; percentage predicted, ppb; parts per billion, RO; Romania, RS; Serbia, SE; Sweden, SI, Slovenia, TR; Türkiye.

### Comorbidity burden

Comorbidities were defined according to local norms. Not all of the 23 comorbidities captured across SHARP were recorded in every country (Supplementary Figure S1). Sinonasal disease was the most prevalent comorbidity in eight countries, and obesity, bronchiectasis, and gastro-oesophageal reflux disease (GORD) were most prevalent in one country each. Nevertheless, considerable heterogeneity in individual comorbidity prevalence was observed by country (see Supplementary Results and Supplementary Figure S1).

### Multimorbidity burden and clustering of comorbidities

Across countries, we observed a median of three comorbidities per patient, in addition to severe asthma (Fig. 1b). Between 87 and 100% of patients had at least one additional comorbidity (Fig. 1c). Countries were grouped into four geographical regions (Fig. 1d and Supplementary Table S1) and a total of ten comorbidities had a ≥5% prevalence in all four regions (Supplementary Table S3). These were: osteoporosis, steroid-induced weight gain, eczema, rhinitis, chronic sinusitis, nasal polyps, obesity, GORD, bronchiectasis, and psychological comorbidities. Substantial heterogeneity in prevalence of these comorbidities was noted by region (Supplementary Table S3).

**Fig. 2:**
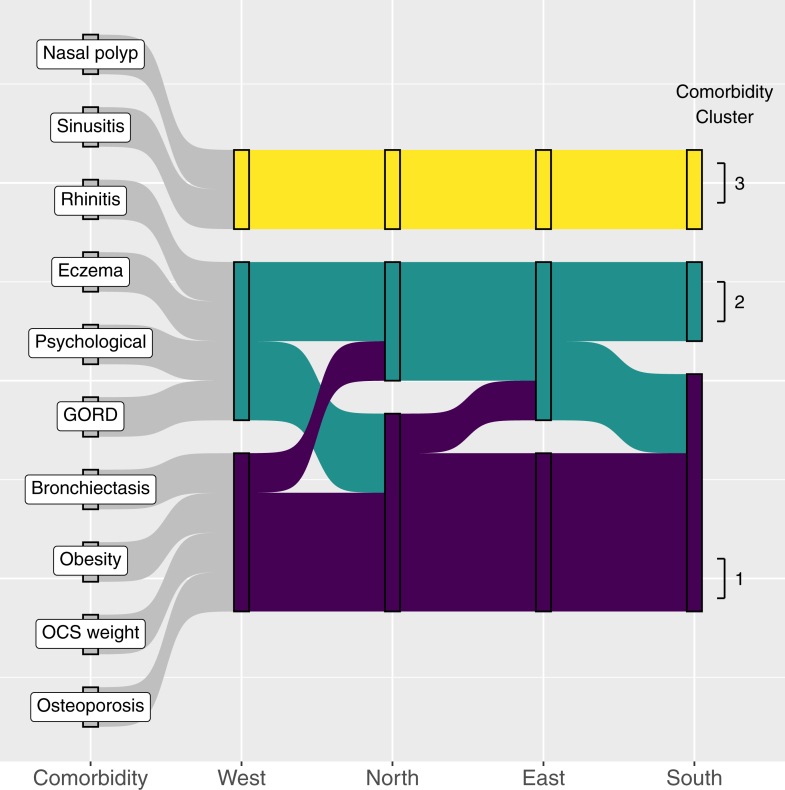
**Comorbidity Clusters as defined by geographical region.** Abbreviations GORD; gastroesophageal reflux disease, OCS weight; oral corticosteroid related weight gain, Psychological; depressive disorder or steroid-induced depression.

Three pairs of ‘anchor’ comorbidities clustered together in all four European regions (Fig. 2, Supplementary Figures S2, and S3). These were Cluster 1: osteoporosis and steroid induced weight gain; Cluster 2: eczema and rhinitis and Cluster 3: chronic sinusitis and nasal polyps (Fig. 2). The four remaining comorbidities (obesity, bronchiectasis, GORD, and psychological comorbidities) localised to Cluster 1 or Cluster 2, depending on European region (Fig. 2). None of the latter co-aggregated with Cluster 3 in any European region. In what follows, we term these four as ‘fluidly-assigned’.

### Patient assignment by multimorbidity phenotypes

**Fig. 3:**
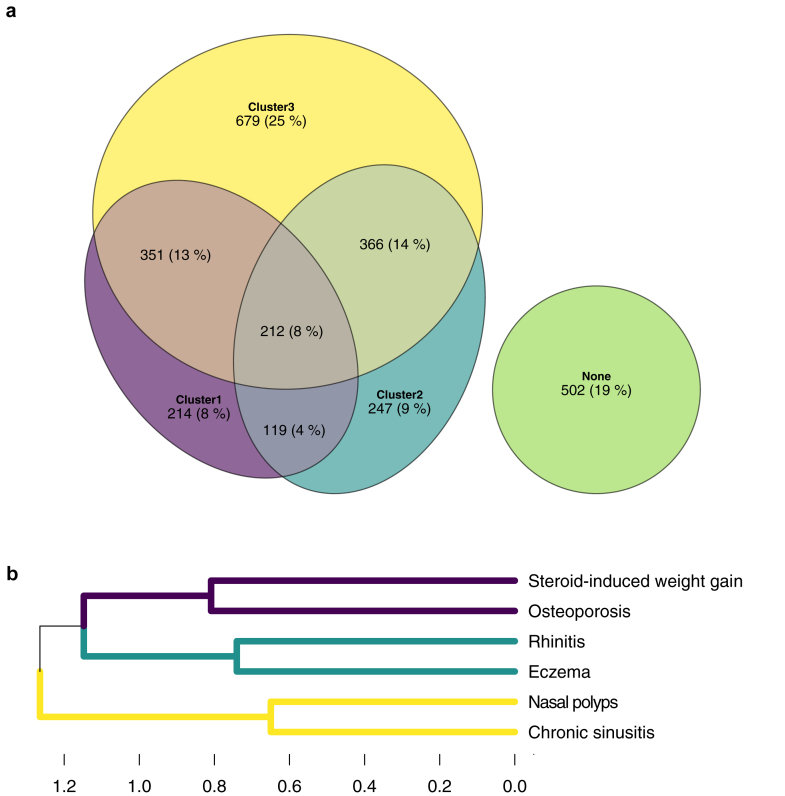
**Distribution of patients according to the presence of comorbidities in 0, 1, 2 or all 3 comorbidity clusters.** a) Euler diagram of patient counts according to their multimorbidity profile. b) The three comorbidity clusters that were consistently detected across all four regions of Europe. Purple denotes cluster 1; green denotes cluster 2; yellow denotes cluster 3.

It was observed that individual patients might exhibit comorbidities in 0,1,2, or all 3 clusters (Fig. 3). Each patient was therefore assigned to a multimorbidity phenotype (MMP) according to whether they had none of the six highlighted anchor comorbidities (MMP u [unclustered multimorbidity]), comorbidities from a single cluster (MMP ster [steroid-associated multimorbidity], MMP all [allergy-associated multimorbidity], or MMP sn [sinonasal-associated multimorbidity]), comorbidities from two clusters (MMP ster-all [steroid + allergy-associated multimorbidity], MMP ster-sn [steroid + sinonasal-associated multimorbidity], or MMP all-sn [allergy + sinonasal-associated multimorbidity]), or comorbidities from all three clusters (MMP123 max; maximal multimorbidity). Geographical heterogeneity was evident for each MMP group (Table 2). MMP sn was the most prevalent for seven countries, and MMP u was most prevalent in the remaining four.

**Table 2:** Multimorbidity phenotypes by country n (%).

Country	MMP u N = 502	MMP ster N = 214	MMP all N = 247	MMP sn N = 679	MMP ster-all N = 119	MMP ster-sn N = 351	MMP all-sn N = 366	MMP max N = 212
Croatia (HR)	24 (11%)	17 (8%)	13 (6%)	45 (21%)	11 (5%)	43 (20%)	25 (12%)	37 (17%)
Hungary (HU)	6 (4%)	2 (1%)	17 (12%)	43 (31%)	14 (10%)	30 (21%)	14 (10%)	14 (10%)
Latvia (LV)	4 (8%)	6 (12%)	3 (6%)	25 (48%)	3 (6%)	4 (8%)	5 (10%)	2 (4%)
Lithuania (LT)	49 (46%)	6 (6%)	6 (6%)	23 (22%)	2 (2%)	8 (8%)	9 (9%)	3 (3%)
Netherlands (NL)	209 (17%)	116 (9%)	78 (6%)	302 (24%)	65 (2%)	201 (16%)	180 (14%)	119 (9%)
Poland (PL)	90 (26%)	21 (6%)	81 (24%)	81 (24%)	10 (3%)	4 (1%)	46 (13%)	12 (4%)
Romania (RO)	16 (42%)	1 (3%)	3 (8%)	12 (32%)	0 (0%)	4 (11%)	2 (5%)	0 (0%)
Serbia (RS)	39 (23%)	11 (7%)	20 (12%)	55 (32%)	2 (1%)	14 (8%)	26 (15%)	3 (2%)
Slovenia (SI)	38 (20%)	26 (14%)	18 (9%)	36 (19%)	10 (5%)	29 (15%)	23 (12%)	13 (7%)
Sweden (SE)	19 (19%)	3 (3%)	6 (6%)	36 (36%)	0 (0%)	9 (9%)	24 (24%)	3 (3%)
Türkiye (TR)	8 (13%)	5 (8%)	2 (3%)	21 (34%)	2 (3%)	5 (8%)	12 (20%)	6 (10%)

**Abbreviations:** MMP; multimorbidity phenotypes.

Clinical phenotypes: MMP u – Unclustered multimorbidity, MMP ster – Steroid-associated multimorbidity, MMP all – Allergy-associated multimorbidity, MMP sn- Sinonasal-associated multimorbidity, MMP ster-all – Steroid + allergy-associated multimorbidity, MP ster-sn – Steroid + sinonasal-associated multimorbidity, MP all-sn – Allergy + sinonasal-associated multimorbidity, MP max – maximal multimorbidity.

### Clinical traits of multimorbidity phenotypes

**Fig. 4:**
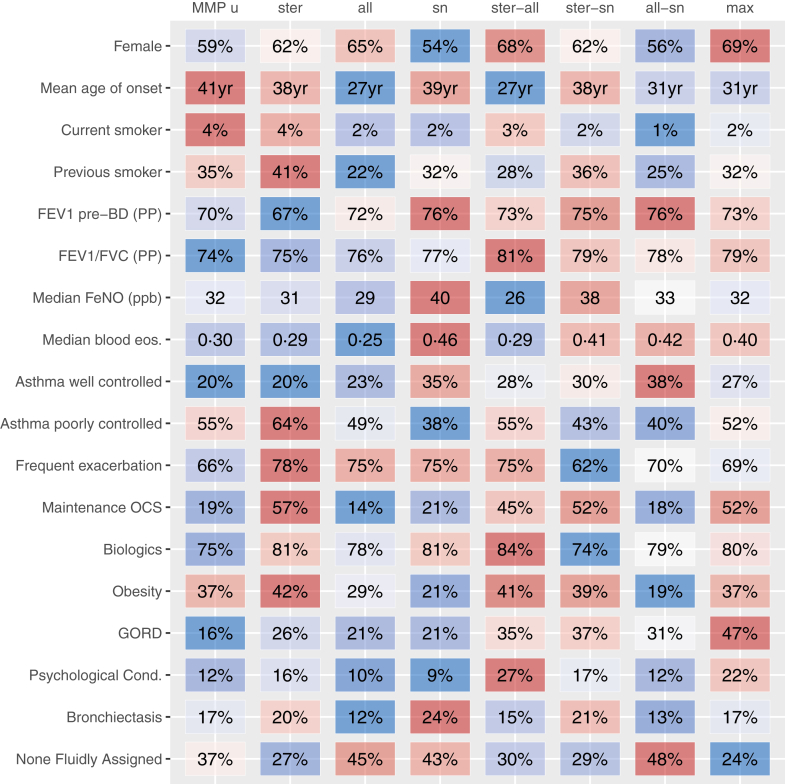
**Key patient characteristics by multimorbidity phenotype.** Values are colour coded, blue to red to indicate low to high. Abbreviations BD; bronchodilator, blood eos.; blood eosinophils (10^9^/L), FeNO, fractional exhaled nitric oxide, FEV_1_; Forced Expiratory Volume in 1 s, FVC; forced vital capacity, MMP; multimorbidity phenotype, OCS; oral corticosteroids, PP; percentage predicted, ppb; parts per billion, Psychological Cond.; Psychological Condition (depressive disorder or steroid-induced depression). Statistical tests of heterogeneity (chi-squared/Wilcoxon) gave p < 0.001 for all rows except FEV1/FVC (%) and Biologic, for which both p = 0.029. Clinical phenotypes: MMP u—Unclustered multimorbidity, MMP ster—Steroid-associated multimorbidity, MMP all—Allergy-associated multimorbidity, MMP sn- Sinonasal-associated multimorbidity, MMP ster-all—Steroid + allergy-associated multimorbidity, MMP ster-sn—Steroid + sinonasal-associated multimorbidity, MMP all-sn—Allergy + sinonasal-associated multimorbidity, MP max—maximal multimorbidity.

Different MMPs displayed numerous distinctive clinical traits (Fig. 4) while the four fluidly-assigned comorbidities were variably present across all MMPs. Biologic treatment was consistently high across MMPs (74%–84%). Steroid related side effects were variable, with higher levels seen in MMP max (Supplementary Table S4).

Patients in MMP u (unclustered multimorbidity; 19%) had latest asthma onset, were more likely to currently smoke, have lower lung function, and poorly controlled asthma. Their use of m-OCS and biologic treatments were low. Despite not demonstrating anchor comorbidities, they were not necessarily comorbidity-free. Only 37% of MMP u demonstrated none of the ten most prevalent comorbidities; 37% had obesity, 17% bronchiectasis, 12% psychological comorbidity, and 16% GORD. Whilst obesity was high, rates of other steroid related comorbidities were low.

Forty two percent of patient phenotypes were categorised by a single comorbidity cluster. Patients in MMP ster (8% of all patients) had high smoking histories, highest m-OCS use, highest rate of frequent exacerbation (≥2 in prior year), worst asthma control and lung function plus highest prevalence of obesity and any of the fluidly assigned comorbidities. They showed low T2-traits (blood eosinophil counts [BEC] and Fractional Exhaled Nitric Oxide [FeNO]). Patients in MMP all (9%) had early-onset asthma, were less likely to smoke or be on m-OCS, with lower rates of steroid related comorbidities, and had lower T2-traits and fewer asthma exacerbations. MMP sn (25%) was the commonest single comorbidity group and had older age of asthma onset, highest male prevalence, highest T2-traits, better lung function, lowest rates of poor asthma control and of taking m-OCS, plus lower rates of steroid related comorbidities. Patients in both MMP all, and MMP sn had lower obesity prevalence and were more likely to have none of the fluidly-assigned comorbidities.

Thirty one percent of patients were categorised by membership of two comorbidity clusters. MMP ster-all showed strong female predominance, earlier onset asthma, low T2-traits, spirometry with a normal ratio, highest biologic use, and high prevalence of obesity plus other fluidly assigned comorbidities. MMP ster-sn patients demonstrated better spirometry, greater T2 inflammatory indices and m-OCS treatment plus high prevalence of obesity and other fluidly assigned comorbidities. MMP all-sn patients had the lowest rates of current smokers, better FEV_1_, low m-OCS use, lowest obesity, were more likely to have no fluidly assigned comorbidities and well controlled asthma.

Lastly, MMP max (8%) comprised more female patients, with high levels of m-OCS and higher BEC. High levels of fluidly-assigned and steroid related comorbidities were present in this group.

## Discussion

To our knowledge, this SHARP study provides the first characterisation of multimorbidity phenotypes and their associated traits in severe asthma. These phenotypes were derived in a stepwise approach. First, we followed an unbiased clustering approach using the ten commonest comorbidities present across all four regions of Europe. Patients were then assigned to multimorbidity phenotypes according to cluster alignment. Two phenotypes, 1) sinonasal-associated (MMP sn) and 2) an unclustered phenotype (MMP u) were consistently commonest in the different European countries. Greatest morbidity was observed in a steroid-associated phenotype (MMP ster), a maximal multimorbidity phenotype (MMP max) and the unclustered phenotype (MMP u). Conversely, the sinonasal-associated multimorbidity phenotype (MMP sn) showed a more airway-centric characterisation and better asthma control. These findings complement the recently described multimorbidity in difficult asthma score (MiDAS).^8^ That score clearly showed the detrimental association of multimorbidity in difficult asthma. MiDAS also highlighted which comorbidities (obesity, bronchiectasis, breathing pattern disorder, GORD, non-steroidal anti-inflammatory disease, sleep apnoea) were most relevant to those effects. It potentially identifies those difficult asthma patients most at risk of adverse asthma and general health outcomes from their multimorbidity. This is clinically relevant since treating multimorbidity in difficult asthma can improve patient outcomes.^5^ The present study advances that understanding by revealing the granular patterns of multimorbidity in severe asthma that could be detected by clinicians (Supplementary Figure S6). In turn, it raises possible avenues for addressing them in a targeted or trait-specific manner.

This study using the SHARP Central database identified extremely high prevalence of multimorbidity in severe asthma. This association with multimorbidity was demonstrated across 11 European countries despite marked variation in individual comorbidity prevalence between countries. Cluster analysis identified three pairs of anchor comorbidities consistently co-aggregated across the four European regions studied: 1) osteoporosis plus steroid-induced weight gain, 2) eczema plus rhinitis, and 3) chronic sinusitis plus nasal polyps. These novel comorbidity clusters are biologically plausible, and replicated across the four defined geographical regions in Europe. The remaining four fluidly-assigned comorbidities (obesity, GORD, bronchiectasis, and psychological comorbidity) clustered variably by geographical region.

Despite considerable variability in individual comorbidity prevalence across European regions, we observed consistent replicability of multimorbidity phenotypes across those same regions. Variability in comorbidity prevalence may reflect regional differences in genetic, epigenetic, behavioural/cultural and environmental factors.^15^ This heterogeneity may also reflect differing collection of comorbidity data, possibly influenced by diverse physician perceptions, clinical recognition and awareness of multimorbidity and available multidisciplinary team (MDT) support. Our work suggests that future standardisation and expansion of comorbidity recording within severe asthma treatment pathways is warranted and may facilitate better asthma management and outcomes. In SHARP, a planned Clinical Protocol for use by participating countries should facilitate better future data harmonisation.

Whilst other national and international registries have explored clustering approaches to phenotype severe asthma,^2^^,^^16^, ^17^, ^18^ these studies have approached severe asthma from an airway centric perspective. As the first study to cluster severe asthma using comorbidities, we bring a novel addition to the literature that provides a holistic view encompassing multimorbidity in severe asthma.

Several of the MMPs identified in this study merit particular clinical awareness. MMP ster might be regarded as a potentially iatrogenic steroid-associated multimorbidity group. Any of the MMPs carrying cluster 1 demonstrated higher asthma related morbidity. In relation to MiDAS, these phenotypes can be anticipated to score higher and experience worse asthma outcomes. Greater exposure to OCS is known to be associated with higher levels of OCS-related comorbidities in asthma patients.^19^ Our steroid-associated MMPs provide the first demonstration of such associations in a multimorbidity context. We also novelly demonstrate their alignment in such a framework with numerous worse asthma severity outcomes despite high steroid and biologic treatment use. Their high disease burden and worse lung function despite biologic therapy is a cause for concern. It has been shown that comorbidities and longer disease duration prior to commencing biologics can be associated with less biologic-induced severe asthma remission. It may be that steroid-associated multimorbidity phenotypes are especially susceptible to such effects.^7^ Indeed, such multimorbidity phenotypes and their clinical features may partly reflect the effect of delayed intervention with targeted biologics.^20^ Additionally, their lower T2-traits may reflect OCS suppressant effects rather than “non-T2 status”, since most severe asthma demonstrates T2 signals.^21^^,^^22^ Conversely, some of these patients may actually have a “symptom high, biomarker low” state less suited to T2-targeted treatments but potentially in need of more holistic (and less airway-centric) assessment and management of their symptoms. Regardless of that point, this overall phenotype, and its associated morbidity, might be prevented by avoidance of OCS over-reliance in future clinical management. This could be either earlier consideration of biologic treatments where clinically appropriate, and/or focus on other comorbidity management as directed by a tool like MiDAS.^23^

MMP ster-all also showed high morbidity. They may represent an early onset, atopic phenotype,^24^^,^^25^ with their comorbidity burden partly reflecting their disease duration and its secondary health characteristics. Lower airway-centric T2-traits may partly reflect treatment suppressive effects, but also distinct inflammatory pathways associated with a more allergic tendency. Their residual disease morbidity raises potential need to target such comorbidities as a means to further improve their clinical outcomes.

MMP max had the greatest collective (both anchor and fluidly assigned) comorbidity burden. MMP max were most likely to be female, with poorer asthma control and higher BEC despite higher m-OCS and biologic use. Steroid related comorbidities were high in this MMP. In relation to MiDAS, this phenotype can be expected to score highly and experience a high level of associated adverse health outcomes. This implies a group of patients for whom T2 high disease persists despite either biological treatment and/or steroids, and who suffer substantial multimorbidity, and for whom novel multimodal treatment approaches beyond those currently available are likely to be needed.^26^

Sinonasal-associated MMPs were common, with MMP sn being the most prevalent phenotype in seven countries. Patients in MMP sn and MMP all-sn showed a more benign status; perhaps unexpected given their T2 high status but homologous with the concept of late onset T2 high, airway-centric disease.^24^ They showed greater well controlled asthma, strong T2-traits, and were least likely to be smokers, with preserved lung function. This perhaps reflects a disease phenotype that responds well to conventional T2-focused airway treatments. That concept aligns with recent demonstration that both chronic rhinosinusitis and nasal polyps are associated with better biologic responses in severe asthma.^27^ We extend those findings by showing that still to be the case when viewed under a wider multimorbidity perspective. A narrower multimorbidity profile would align with lower MiDAS and better clinical outcomes. Apart from bronchiectasis, they showed lower levels of the fluidly assigned comorbidities. Contrastingly, MMP ster-all (steroid + sinonasal-associated) had high prevalence of both obesity and GORD accompanied by high m-OCS but lowest biologic use, high T2-traits and poorer asthma control. This could represent a subtype of more severe sinonasal disease that ends up being treated with excessive m-OCS. This, in turn, may facilitate a multimorbidity model with obesity and GORD that collectively sustains a more severe asthma phenotype. Their MiDAS would be expected to be worse. Their treatment may benefit from earlier biologic access alongside management of the components of their multimorbidity via a personalised precision medicine approach.

Four of the comorbidities assessed in our initial clustering analysis did not cluster consistently with other comorbidities across the four European regions. At least one of these fluidly assigned comorbidities occurred in over 50% of each MMP. These fluidly assigned comorbidities, namely bronchiectasis,^28^ obesity,^7^ GORD,^28^ and psychological comorbidity,^28^^,^^29^ have all individually shown association with adverse health outcomes in severe asthma. Furthermore, both bronchiectasis and GORD have been shown to be independent predictors of poor disease control in severe asthma over a 5-year follow-up period.^3^ Additionally, obesity, GORD, and bronchiectasis are all associated with worse asthma severity status within MiDAS. They therefore strongly merit screening for, and treatment when present, in severe asthma patients.

Obesity merits particular attention among the fluidly assigned comorbidities. It showed highest prevalence in the MMPs with highest m-OCS use (MMP ster, MMP ster-all, MMP ster-sn, MMP max) suggesting corticosteroid association. In this context, steroid induced weight gain could be viewed as both an iatrogenic consequence of treatment with OCS as well as a comorbidity arising from that exposure. However, obesity prevalence was also high (37%) in MMP u, who reported low m-OCS use and low rates for other steroid-related comorbidities, demonstrating that obesity in severe asthma is likely to be multifactorial. Indeed, the association of asthma and obesity is bidirectional. Asthma itself has been demonstrated to be associated with a higher risk of developing obesity, particularly in those who are non atopic, with longer disease duration and on OCS.^30^ This is noteworthy since MMP u was the most prevalent multimorbidity phenotype in four countries. Such findings extend those from ISAR, where obesity was not significantly associated with higher m-OCS use.^2^ An intriguing novel concept is of persistent T2 metabolic-driven inflammation, with data emerging that obesity is associated with T2 inflammation in asthma.^31^ Given negative impacts of obesity on asthma biologic responses, this subset of T2 inflammation may be less responsive to current biologic treatments.^7^ MiDAS demonstrated association of worse multimorbidity with T2 cytokines, which are also higher in obesity, highlighting complex interrelationships that require further research. It is important to note that our present cross-sectional analysis cannot explore potential confounding by access to biologic treatments by country and subsequent impact on T2 signal, asthma outcomes, steroid use and steroid side effects. Future, planned work will seek to investigate such temporal associations along with longitudinal outcomes, by phenotype. Regardless, our findings of prominent association between obesity and multiple worse outcome MMPs further emphasises the need to actively address obesity management in severe asthma patients.

Strengths of this work include the large number of patients across a wide geographical area in Europe, and employment of an unbiased clustering analysis approach. Our sizeable study population reflected the nature of severe asthma patients encountered in real-world settings across multiple countries and therefore was representative of patients routinely encountered in clinical practice. The analyses, grouped into four geographical regions, offer internal replication within the SHARP dataset, mitigating criticisms about lack of external replication. Similar to any central registry, we are limited by the extent of the data captured in the national registries and their retrospective nature. It is also noted that data are from a number of different registries. Kroes et al. describe how these data are harmonised into the Observational Medical Outcomes Partnership Common Data Model to allow analyses across registries.^32^ Biological measures and clinical status measures were not always from contemporaneous timepoints in registries. National (country-specific) reimbursement/eligibility criteria for biologics might have meaningful impact on severe asthma patient characteristics captured in respective national registries. Thus, observed differences linked to OCS-related comorbidities may, in part, result from local health systems and severe asthma network specificities including referral criteria. Comorbidity data capture may be influenced by local prevalence, but also by physician bias, the availability of local diagnostics and access to a broader MDT with the skills to address these comorbidities, when identified. This is particularly relevant to the omission of cardiovascular comorbidities, aspirin sensitivity and breathing pattern disorder. These would have been important to include but were not available in the dataset. Future asthma research needs to assess for such wider comorbidities. We acknowledge the limitation that in our study, the West of Europe data was drawn solely from the Netherlands. We, therefore, may not have captured the full diversity of severe asthma multimorbidity within the region.

The range of West European countries were limited only by engagement from other countries in that region. However, it should also be noted that the Dutch National Registry was the largest contributing national dataset, ensuring a substantial representation from the West of Europe in our analysis. That non uniform sampling, in turn, may be viewed as a potential study limitation. Yet, despite this fact we saw consistent findings across the different regions of Europe supporting their wider relevance and generalisability. Ethnicity data was also not collected in SHARP and future work should assess whether multimorbidity phenotypes are influenced by factors such as ethnicity. A further potential limitation is that we have confined our present analysis to cross-sectional data. That in part reflects the need to initially identify and comprehensively characterise these novel multimorbidity phenotypes for the first time. Furthermore the SHARP dataset for this study currently has limited longitudinal outcome data availability. These combined considerations place inclusion of longitudinal outcomes beyond the scope of the present paper. Future planned work will therefore investigate longitudinal outcomes, by phenotype. Another limitation is the lack of data on hospital admissions and quality of life for these multimorbidity phenotypes. While that would have been very informative it was unfortunately not available in the current SHARP dataset. That dataset though did have data on a wide range of outcome domains including asthma control, exacerbations, lung function and m-OCS needs. Therefore, meaningful multidimensional understanding of phenotype severity was still possible. The SHARP dataset also lacked certain parameters (non-steroidal anti-inflammatory exacerbated respiratory disease and breathing pattern disorder) core to MiDAS. This meant that we could not directly assess our multimorbidity phenotypes against MiDAS. Nevertheless we were still able to broadly estimate their anticipated alignment with MiDAS. Finally, there is potential for cross-over between comorbidity labels; for example, chronic sinusitis and rhinitis may be used interchangeably by some. However, these are clinically distinct entities, with different aetiologies^33^ and the comorbidity categories used in SHARP are sufficiently distinct to infer identification of separate comorbidities.

In conclusion, we have demonstrated that most patients with severe asthma experience multimorbidity and that there are distinct multimorbidity phenotypes in severe asthma with different adverse outcome risks. Previously, understanding of multimorbidity in severe asthma was largely based on clinical intuition. The current study helps fill that knowledge gap. The findings provide an important evidenced reference point from which clinicians can better address the full needs of their patients with severe asthma. The identification of MMPs with worse outcomes highlight the importance of holistic severe asthma management, addressing multimorbidity burden alongside T2 inflammation, and offer structure to assessment of multimorbidity within the clinic. The fact that some of the more severe MMPs were steroid-associated emphasises the need to eradicate oral steroid dependency in severe asthma management. It guides clinical thinking towards earlier consideration of biologic therapy where that is most appropriate. It also highlights need for a considered approach to understand more complex symptom presentations where factors beyond airway pathophysiology are likely to be relevant. Given globally rising rates of obesity^34^ and associated metabolic dysfunction,^35^ diabetes,^36^ anxiety and depression,^37^ multimorbidity will remain a burden for patients with severe asthma independent of OCS usage. Alongside insights provided by MiDAS,^8^ identification of multimorbidity phenotypes that are stable across geographical regions, offer structure and clinically applicable frameworks, to take recognition, assessment and management of severe asthma to another level of precision medicine. That may not only deliver patient-level but health economic benefits through optimised resource use and improved patient outcomes.^23^ Ultimately such better multimorbidity-based phenotypic understanding may also facilitate new more patient-centred and holistic treatment models for patients with severe asthma to better address and improve “whole patient” outcomes.

## Contributors

RJ Kurukulaaratchy (RJK), A Freeman (AF), Saša Rink (SR) and Aruna Bansal (ATB) developed the initial study concept and undertook preliminary analyses. RJK, AF, SR, ATB, Florence Schleich (FS), Betty Frankemölle (BF), Mehar Singh (MS), Jacob Sont (JKS), Ben Ainsworth (BA), Apostolos Bossios (AB), Michael Hyland (MH), Dace Matisa (DM), and Sabina Škrgat (SS) evolved subsequent study design and methodology. ATB was responsible for data curation and formal analysis to create comorbidity clusters and multimorbidity phenotypes. ATB, AF, and RJK directly accessed and verified the underlying SHARP study data reported in the manuscript. AF and RJK wrote the original draft with visualisations provided by ATB. RJK, AF, SR, ATB, FS, BF, MS, JKS, BA, AB, MH, DM, SS, Rekha Chaudhuri, Florin Milhaltan, Antonio Spanevello, Enrico Heffler, Ian Adcock, Martina Zappa, Giorgio Walter Canonica, Guy Brusselle, Arnaud Bourdin, Giulia Anna Maria Luigia Costanzo, Ildiko Horvath, Dóra Lúðvíksdóttir, Stefania Principe, Peter Kopač, Cláudia Chaves Loureiro, Salman Siddiqui, Arne Egesten, Virginija Kalinauskaite-Zukauske, Sanja Dimic-Janjic, Graham Roberts, Sanja Hromis, Branislava Milenkovic, Judit Varkonyi-Sepp, Ozlem Goksel, Ana M Pereira, Ratko Djukanovic, Angela Rizzi, Marco Caminati, Ruihua Hou, Anamarija Štajduhar, Dóra Paróczai, Luisa Brussino, Liam Heaney, Hans Michael Haitchi, Matteo Bonini, Kristina Bieksiene, Ebru Damadoglu, Valentyna Yasinska, Bilun Gemicioglu, Sanja Popović Grle, Anneke ten Brinke, Zsuzsanna Csoma, Iveta Kroica, Piotr Kuna, Barbro Dahlen, Celeste Porsbjerg, and Hilary Hodge all contributed to study conceptualisation, data interpretation and study writing. All authors reviewed and edited the draft manuscript before also approving the final version for submission. RJK acts as guarantor for the work and held final responsibility for the decision to submit the article.

## Data sharing statement

After publication, the data will be made available to others on reasonable requests to the corresponding authors. A proposal with detailed description of study objectives and statistical analysis plan will be needed for evaluation of the reasonability of requests. Additional materials might also be required during the process of evaluation. Deidentified participant data will be provided after approval from the corresponding authors and the SHARP steering committee.

## Declaration of interests

RJ Kurukulaaratchy co-holds a methods patent outside the submitted work on the cellular profiles of Tissue Resident Memory T-cells and their use in asthma.

B Ainsworth is a member of the UK Taskforce for Lung Health, has received honoraria for educational talks from AstraZeneca, and sits on advisory boards for the Medito Foundation and earGym. All of these are unrelated to this work.

R Djukanovic is a Past co-Chair of the European Respiratory Society's Clinical collaboration on severe asthma ∗SHARP. He is also a co-founder and shareholder of and consultant to Synairgen, has received funding for lectures from GlaxoSmithKline, and has been on advisory boards of GlaxoSmithKline, Celltrion, ALK Abello and ZenasBio, all unrelated to this work.

S Hromis reports speakers fees from AstraZeneca, Berlin Chemie Menarini, Takeda, Providens, Amicus Therapeutics; support for attending meetings from AstraZeneca, Chiesi (Providens), Hemofarm and payment for advisory boards from AstraZeneca, BerinChemie Menarini, Providens. These were all unrelated to this work.

HM Haitchi co-holds a method patent on Anti-ADAM33 oligonucleotides and related methods, which is unrelated to the current work.

I Adcock reports institutional grants from GlaxoSmithKline, MRC, Sanofi, and EPSRC; consulting fees from GlaxoSmithKline and Kinaset; funding for lectures from AstraZeneca and GlaxoSmithKline, and has served on advisory boards for GlaxoSmithKline, Sanofi, Chiesi and Kinaset. All unrelated to this work.

M Florin has received funding for lectures with presentations from AstraZeneca, Sanofi, Pfizer, and Angelini, unrelated to this work.

B Gemicioglu is Chair of the Turkish Board of Pulmonology and GARD Turkey Coordinator (unpaid); reports institutional Honoria for lectures from Abdi Ibrahim, AstraZeneca, Daeva, and GlaxoSmithKline; support for attending meetings from AstraZeneca and GlaxoSmithKline and participation on advisory boards of GlaxoSmithKline. Unrelated to this work.

B Dahlén reports personal Honoria for lectures from AstraZeneca, GlaxoSmithKline, and Sanofi, as well as payment for participation on advisory boards of Affibody and is associated with the Swedish Medical Products Agency, all unrelated to this work.

P Kuna reports grants for investigator led academic study from AstraZeneca, payment for lectures from Adamed, GlaxoSmithKline, AstraZeneca, Glenmark, Teva, Polpharma, and Berlin Chemie Menarini, and conference travel grants from AstraZeneca and Berlin Chemie Menarini, all unrelated to this work.

E Damadoglu is Chair of the Turkish Thoracic Society Asthma Section (unpaid), unrelated to this work.

M Caminati reports consulting fees from AstraZeneca and Sanofi and speaker fees from GlaxoSmithKline, AstraZeneca, and Sanofi, all unrelated to this work.

AT Brinke reports research grants from AstraZeneca, GlaxoSmithKline and TEVA, consulting fees/advisory board from AstraZeneca, GlaxoSmithKline, Novartis and TEVA and payment for lectures from AstraZeneca, GlaxoSmithKline, Novartis, TEVA and SanofiGenzyme. all unrelated to this work.

A Egesten participated on advisory boards for BioCryst, and CSL-Behring, unrelated to this work.

C Chaves Loureiro received grants for investigator led study from AstraZeneca and GlaxoSmithKline; consulting fees from AstraZeneca, GlaxoSmithKline, and Sanofi; payment for lecture presentations from GlaxoSmithKline, AstraZeneca, TEVA, and Sanofi; support for attending conferences from AstraZeneca, Sanofi and Vivisol, and served on advisory boards for AstraZeneca, GlaxoSmithKline, Sanofi, and Menarini. All unrelated to this work.

GAML Costanzo received support for conference attendance from Chiesi, Sanofi, GlaxoSmithKline, and AstraZeneca, unrelated to this work.

G Roberts is the Past president of the British Society of Allergy and Clinical Immunology and reports grants from the EU 3TR Consortium and consulting fees from AstraZeneca, not related to this work.

G Brusselle is President of the Belgian Respiratory Society (BeRS) and reports speaker fees from AstraZeneca, Boehringer-Ingelheim, Chiesi, GSK, Novartis, Merk Sharp & Dohme and Sanofi Regeneron. All unrelated to this work.

J Varkonyi-Sepp reports research grants from GlaxoSmithKline, separate to this work.

K Bieksiene received lecture fees from AstraZeneca, Berlin Chemie, and Chiesi and support for conference and scientific meeting attendance from Chiesi, AstraZeneca, and Berlin Chemie, all not related to this work.

M Zappa reports payment for lectures from AstraZeneca, unrelated to this work.

O Goksel received grants for investigator led study from AstraZeneca, GlaxoSmithKline, and Sanofi as well as payment for lectures from GlaxoSmithKline not related to this work.

P Kopač reports consulting fees from AstraZeneca, Berlin-Chemie Menarini, Medis, Swixx Biopharma; speaker fees from AstraZeneca, Berlin-Chemie Menarini, Medis, and Swixx Biopharma; and support for attending conferences from AstraZeneca, and Berlin-Chemie Menarini, all unrelated to this work.

R Chaudhuri received grants for investigator led study from AstraZeneca; payment for lectures from GlaxoSmithKline, AstraZeneca, Teva, Chiesi, and Sanofi; support for attending conferences from Chiesi, Sanofi, and GlaxoSmithKline; and participation on advisory boards of GlaxoSmithKline, AstraZeneca, and Celltrion. All unrelated to this work.

S Dimic-Janjic reports payment for lectures from Boehringer Ingelheim, AstraZeneca, Berlin Chemie Menarini, Takeda, and Providens and participation on advisory boards of Boehringer Ingelheim, AstraZeneca, Berlin Chemie Menarini, and Takeda. All unrelated to this work.

C Porsbjerg reports institutional grants from AstraZeneca, GlaxoSmithKline, Novartis, TEVA, Sanofi, Chiesi, and ALK; consulting fees and payment for lectures (paid to institution and personal Honoria) from AstraZeneca, GlaxoSmithKline, Novartis, TEVA, Sanofi, Chiesi, and ALK; and participation on advisory boards of AstraZeneca, Novartis, TEVA, Sanofi, ALK. All unrelated to this work.

A Spanevello reports grants from GlaxoSmithKline, Sanofi, and Menarini; consulting fees from Chiesi and Sanofi; payment for lectures from GlaxoSmithKline, AstraZeneca, Chiesi, and Sanofi; support for attending conferences from Chiesi, Sanofi and GlaxoSmithKline; and sits on advisory board meetings of GlaxoSmithKline and Sanofi. All unrelated to this work.

S Principe reports grants from Innovative Medicines Initiative 2 Joint Undertaking (JU) and the European Union's HORIZON Research and Innovation programme, and is associated with the Young Investigator Board—Netherlands Respiratory Society (unpaid) All unrelated to this work.

V Kalinauskaite-Zukauske reports payment for lectures from AstraZeneca, Chiesi, Sanofi, and Medison as well as support for attending conferences and scientific meetings from Chiesi, AstraZeneca, and Medison. All unrelated to this work.

V Yasinska reports grants for investigator led study from AstraZeneca; payment for lectures from GlaxoSmithKline, AstraZeneca, and Sanofi; and participation on Advisory boards (payment to institution) of GlaxoSmithKline and AstraZeneca, all unrelated to this work.

Z Csoma reports grants for investigator led study from AstraZeneca; payment for lectures from AstraZeneca, Chiesi, Sanofi, and Belin Chemie; support for attending scientific meetings from Orion Pharma and participation on Chiesi and AstraZeneca advisory boards, all unrelated to this work.

AM Pereira received support for attending scientific meetings from Menarini and Roxall, not related to this work.

A Štajduhar received support for attending scientific meetings from AstraZeneca unrelated to this work.

D Paróczai reports personal university research grants from the University Research Fellowship Program (EKÖP) of the Ministry for Culture and Innovation from the source of the National Research, Development and Innovation Fund (EKÖP-24-4–SZTE-376) and ÚNKP-23-4-New National Excellence Program of the Ministry for Culture and Innovation, the National Research, Development, and Innovation Fund (ÚNKP-23-4-SZTE-380), unrelated to this work.

E Heffler reports grants from Chiesi paid to his institution; personal consultancy fees from Chiesi, GlaxoSmithKline, Sanofi, AstraZeneca, Regeneron, Almirall, Apogee Therapeutics, Celltrion Healthcare, and Bosch; payment for lectures from Chiesi, GlaxoSmithKline, AstraZeneca, Sanofi, Regeneron, Novartis, Lofarma, and Firma; and participation on GlaxoSmithKline, AstraZeneca, and Sanofi advisory boards. All unrelated to this work.

R Hou participates on advisory boards for the Medical Research council, ECNP and AAIC NPI (programme chair). All unrelated to this work.

LG Heaney has received institutional project grant funding from AstraZeneca and GlaxoSmithKline and has been involved in asthma clinical trials with GlaxoSmithKline, AstraZeneca and Roche/Genentech for which his institution was renumerated. He has given lectures supported by AstraZeneca, Sanofi, Circassia, GlaxoSmithKline, and Teva; received travel support to attend international respiratory meetings from AstraZeneca, Sanofi, Teva and GSK; and attended advisory boards/lectures of GlaxoSmithKline, AstraZeneca, and Celltrion. Unrelated to this work.

A Bourdin reports grants from AstraZeneca, Boeringher Ingelheim, and GlaxoSmithKline; consulting fees from AstraZeneca, GlaxoSmithKline, Sanofi, Chiesi, Celltrion, Boeringher Ingelheim, and Novartis; speaker fees from Sanofi Regeneron, AstraZeneca, GlaxoSmithKline, Boeringher Ingelheim, and Novartis; support for attending scientific meetings from AstraZeneca, and Sanofi; and participates on the AB science advisory board, all unrelated to this work.

I Horvath has received personal grants from AstraZeneca and Boeringher Ingelheim; consulting fees from AstraZeneca, Boeringher Ingelheim, Sanofi, and Chiesi; payment for lectures from Sanofi, AstraZeneca, Chiesi, Berlin-Chemie Menarini, and Boeringher Ingelheim; and travel fees from AstraZeneca, Chiesi, Boeringher Ingelheim, and MSD, all unrelated to this work.

S Popović-Grle has received personal consultancy fees from AstraZeneca, Pliva Hrvatska, and Providens as well as payment for lectures from AstraZeneca, Berlin-Chemie, Pliva Hrvatska, and Providens, all unrelated to this work.

A Bossios is Head of Assembly 5 (Airway diseases, asthma, COPD, and chronic cough), European Respiratory Society; co-chair of the Nordic severe asthma network; member of the steering committee of SHARP, ERS severe asthma Clinical Research Collaboration; member of the steering committee of the Swedish National Airway Register. He reports a grant from AstraZeneca as well as Honoraria and lecture fees from Chiesi, GlaxoSmithKline, and AstraZeneca, paid to institution outside of the submitted work. All unrelated to this work.

D Lúdvíksdóttir reports Honoraria for lectures from GlaxoSmithKline, Sanofi and Chiesi; and travel fees from Chiesi, all unrelated to this work.

M Bonini has received research grants and advisory board/speaker fees from AstraZeneca, Boehringer Ingelheim, Chiesi, Grifols, GlaxoSmithKline, Lallemand, Lusofarmaco. Menarini, Omron, and Sanofi, all unrelated to this work.

F Schleich received grants from AstraZeneca, Sanofi, GlaxoSmithKline and Chiesi to give lectures and perform research activities. All unrelated to this work.

JK Sont received Institutional Grants from: AstraZeneca, Dutch RAPSODI severe asthma registry, Care Research Netherlands (ZonMW). All unrelated to this work.

M Hyland declares grants from GlaxoSmithKline and from AstraZeneca outside the submitted work.

GW Canonica reports research or clinical trials grants paid to his Institution from Menarini, AstraZeneca, GlaxoSmithKline, Sanofi Genzyme and fees for lectures or advisory board participation from Menarini, AstraZeneca, CellTrion, Chiesi, Faes Farma, Firma, Genentech, Guidotti-Malesci, GlaxoSmithKline, HAL Allergy, Innovacaremd, Novartis, OM-Pharma, Red Maple, Sanofi-Aventis, Sanofi-Genzyme, Stallergenes-Greer and Uriach Pharma. All unrelated to this work.

S Škrgat reports Honoraria for Educational events, invited lectures and presentations supported by Sanofi, AstraZeneca, Medis, Berlin Chemie, and Chiesi, as well as participation on a local advisory board for AstraZeneca. All unrelated to this work.

S Siddiqui has received fees for advisory service from AstraZeneca, GlaxoSmithKline, Chiesi, Sanofi, Areteia. Speaker fees from AstraZeneca, GlaxoSmithKline, Chiesi, Areteia & Medscape, and is the ERS Clinical Research Collaborations Director. All unrelated to this work.

S Rink has received honoraria for lectures and educational events from Berlin Chemie, Chiesi and Medis and support for attending meetings from AstraZeneca, GlaxoSmithKline, Chiesi and Berlin Chemie. All unrelated to this work.

All other authors have no conflicts of interests to declare.

## Appendix A Supplementary data

Supplementary Materials and Figures

Supplymentary Material
