# Supplementary material for: Multimorbidity phenotypes and associated characteristics in severe asthma: an observational study of European severe asthma registries
Source: Lancet Reg Health Eur. 2026 Feb 5;63:101600. doi: 10.1016/j.lanepe.2026.101600 (PMC12906202; doi:10.1016/j.lanepe.2026.101600)
Supplement: Supplementary Materials and Figures [file mmc1.docx]

**Supplementary Material**

**Title: Multimorbidity phenotypes and associated characteristics in severe asthma: An observational study of European severe asthma registries**

**Table of Contents**

1. **Supplementary Results**
   1. **Demographic data, clinical characteristics and geographical variation Page 2**
2. **Supplementary Tables Page 3-5**
   1. **Supplementary Table 1: Geographical grouping of Countries into North/ South/ East/ West.**
   2. **Supplementary Table 2: Baseline demographics, by region**
   3. **Supplementary Table 3: Comorbidity prevalence by geographical region**
   4. **Supplementary Table 4: Prevalence of steroid related comorbidities across multimorbidity phenotypes.**
3. **Supplementary Figures Page 6-11**
   1. **Supplementary Figure 1: Percentage prevalence of comorbidities by country.**
   2. **Supplementary Figure 2: Dendrogram of comorbidity clustering across the 4 geographical regions (West, North, East, South)**
   3. **Supplementary Figure 3: Dendrogram of consensus comorbidity clustering**
   4. **Supplementary Figure 4: Scree plots, by region**
   5. **Supplementary Figure 5: Item Information curves (IIC).**
   6. **Supplementary Figure 6: Clinical relevance of Multimorbidity Phenotypes**

**Supplementary Results:**

*Demographic data, clinical characteristics and geographical variation*

Patient demographics varied across Europe (table 1) with female predominance of between 51-71% (Sweden vs Latvia). Body mass index (BMI) varied from high normal (24·8) to the high end of overweight (29) (Romania vs Lithuania). Most asthma was adult onset (≥ 18-years of age; 77-97%). Rates of previous/current smoking or pack year history differed across countries with highest current smoking in Serbia and lowest in Sweden, Poland and Latvia. Worst spirometry (median Forced Expiratory Volume in 1 second (FEV_1_)/ Forced Vital Capacity (FVC)) was found in Serbia, with the highest ratio found in the Netherlands. Highest median FeNO was reported in Slovenia and lowest in Romania. The prevalence of atopy (nationally defined) ranged from 21-58% (Latvia vs Serbia). Highest median blood eosinophil counts (BEC) recorded were in Türkiye at 0·7x10^9^/L. Biologic (90% in Netherlands vs 50% in Hungary) and maintenance oral steroid (m-OCS; 48% in Türkiye vs 10% in Lithuania) treatment were strikingly different across countries. Patients in Lithuania, Türkiye, Serbia and Sweden had higher exacerbation frequency, with > 90% of patients in these countries having 2 or more exacerbations per year.

*Comorbidity prevalence across geographical regions*

Comorbidity prevalence differed considerably across countries. Specifically Eosinophilic Granulomatosis with Polyangiitis (EGPA) (0·3-9.8%), nasal polyps (18-45%) and gastroesophageal reflux disorder (GORD) (12-55%) (supplementary figure 1). Sinonasal disease was the most prevalent comorbidity in eight countries, and obesity, bronchiectasis and GORD were most prevalent in one country each (supplementary figure 1). Levels of potentially steroid-related comorbidities (adrenal insufficiency, cataract, diabetes, hypertension, obesity, steroid-induced weight gain, steroid-associated skin disorders, osteoporosis, supplementary table 4) varied greatly across countries and did not mirror rates of m-OCS or exacerbation frequency. Metabolic dysfunction-related comorbidities (diabetes, obesity) varied across regions and did not show parallel prevalence patterns. Cardiovascular disease (ischaemic heart disease, hypertension) prevalence ranged from 2-17%, without demonstrating parity with other metabolic comorbidities, such as diabetes.

Adrenal insufficiency (AI) was highest in Slovenia, which had higher prevalence of patients on m-OCS and with frequent asthma exacerbations but contrastingly high prevalence of well controlled patients. Türkiye, with highest m-OCS, also demonstrated high levels of secondary AI.

**Supplementary Table 1: Geographical grouping of Countries into North/ South/ East/ West.**

| Region | LT | LV | SE | HU | PL | RO | HR | RS | SI | TR | NL |
| --- | --- | --- | --- | --- | --- | --- | --- | --- | --- | --- | --- |
| North | **106** | **52** | **100** |  |  |  |  |  |  |  |  |
| East |  |  |  | **140** | **345** | **38** |  |  |  |  |  |
| South |  |  |  |  |  |  | **215** | **170** | **193** | **61** |  |
| West |  |  |  |  |  |  |  |  |  |  | **1270** |

Abbreviations: HR; Croatia, HU; Hungary, LT Lithuania, LV; Latvia, NL; Netherlands, PL; Poland, RO; Romania, RS; Serbia, SE; Sweden, SI, Slovenia, TR Türkiye

**Supplementary Table 2: Baseline demographics, by region**

|  | North N=258 | East N=523 | South N=639 | West N=1270 | p-value |
| --- | --- | --- | --- | --- | --- |
| Age at index date | 59 (12) | 55 (14) | 55 (13) | 56 (15) | <0.001 |
| Female | 159 (62%) | 345 (66%) | 407 (64%) | 693 (55%) | <0.001 |
| Height (cm) | 169 (10) | 167 (11) | 168 (11) | 172 (11) | <0.001 |
| Weight (kg) | 81 (18) | 78 (18) | 77 (17) | 84 (17) | <0.001 |
| BMI (kg/m^2^) | 28.2 (6.0) | 27.9 (5.7) | 27.4 (5.4) | 28.1 (5.3) | 0.100 |
| Onset of Asthma |  |  |  |  | <0.001 |
| Childhood onset | 36 (15%) | 116 (23%) | 85 (14%) | 312 (27%) |  |
| Adult onset | 206 (85%) | 384 (77%) | 526 (86%) | 836 (73%) |  |
| Smoking History |  |  |  |  | <0.001 |
| Current smoker | 6 (2.4%) | 7 (1.3%) | 37 (5.9%) | 14 (1.1%) |  |
| Previous smoker | 48 (19%) | 68 (13%) | 191 (30%) | 537 (43%) |  |
| Never smoked | 199 (79%) | 447 (86%) | 401 (64%) | 710 (56%) |  |
| Pack years | 10 (5, 22) | 10 (5, 20) | 16 (8, 30) | 11 (5, 22) | <0.001 |
| Clinical Features |  |  |  |  |  |
| Atopy | 78 (30%) | 225 (43%) | 300 (47%) | 606 (48%) | <0.001 |
| FEV_1_ pre-BD as % predicted | 71 (21) | 65 (21) | 68 (20) | 80 (20) | <0.001 |
| FEV_1_ post-BD as % predicted | 72 (20) | 65 (19) | 67 (19) | 84 (20) | <0.001 |
| FEV_1_/FVC as % predicted | 72 (17) | 72 (15) | 68 (15) | 82 (15) | <0.001 |
| FeNO (ppb) | 28 (18, 53) | 32 (19, 52) | 42 (23, 69) | 33 (19, 54) | <0.001 |
| Blood eosinophil count (x10^9^/L) | 0.43 (0.20, 0.70) | 0.31 (0.14, 0.58) | 0.40 (0.20, 0.71) | 0.35 (0.15, 0.66) | <0.001 |
| Asthma control |  |  |  |  | <0.001 |
| Well controlled | 67 (29%) | 92 (22%) | 182 (34%) | NA |  |
| Partly controlled | 41 (18%) | 132 (32%) | 114 (21%) | NA |  |
| Poorly controlled | 122 (53%) | 185 (45%) | 247 (45%) | NA |  |
| >=2 exacerbations / yr | 171 (90%) | 240 (58%) | 474 (81%) | 799 (66%) | <0.001 |
| Biologic treatment | 188 (73%) | 373 (71%) | 413 (65%) | 1,148 (90%) | <0.001 |
| Maintenance OCS, n (%) | 45 (17%) | 78 (15%) | 264 (41%) | 418 (33%) | <0.001 |

Abbreviations: BMI – Body Mass Index, FEV_1_ – Forced Expiratory Volume in 1 second, FVC – Forced Vital Capacity, BD – Bronchodilator, FeNO – Fractional Exhaled Nitric Oxide, OCS – Oral Corticosteroids

**Supplementary Table 3: Comorbidity prevalence by geographical region.**

| Comorbidity | North N=258 | East N=523 | South N=639 | West N=1270 | Heterogeneity  p-value |
| --- | --- | --- | --- | --- | --- |
| Bronchiectasis | 67 (26%) | 46 (9%) | 139 (22%) | 241 (19%) | <0·001 |
| Chronic sinusitis | 126 (49%) | 242 (46%) | 343 (54%) | 689 (54%) | 0·011 |
| Eczema | 18 (7%) | 31 (6%) | 43 (7%) | 249 (20%) | <0·001 |
| Gastroesophageal reflux disease | 57 (22%) | 131 (25%) | 248 (39%) | 277 (22%) | <0·001 |
| Obesity | 76 (30%) | 133 (33%) | 185 (30%) | 385 (30%) | 0·800 |
| Osteoporosis | 24 (9%) | 56 (11%) | 176 (28%) | 284 (22%) | <0·001 |
| Nasal polyps | 105 (41%) | 148 (28%) | 227 (36%) | 482 (38%) | <0·001 |
| Psychological comorbidity | 23 (9%) | 75 (14%) | 79 (12%) | 186 (15%) | 0·069 |
| Rhinitis | 61 (24%) | 201 (38%) | 207 (32%) | 310 (24%) | <0·001 |
| Steroid-induced weight gain | 30 (12%) | 70 (13%) | 99 (15%) | 337 (27%) | <0·001 |

Abbreviations: North (Lithuania (LT), Latvia (LV), Sweden (SE); East (Hungary (HU), Poland (PL) Romania (RO); South (Croatia (HR), Serbia (RS), Slovenia (SL), Türkiye (TR)); West (Netherlands (NL)

**Supplementary Table 4: Prevalence of steroid related comorbidities across multimorbidity phenotypes.** **All rows had p<0.001 in a chi-square test of heterogeneity.**

| OCS Related Comorbidity | MMPu N=502 | MMP ster N=214 | MMP all N=247 | MMP sn N=679 | MMP ster-all N=119 | MMP ster-sn N=351 | MMP all-sn N=366 | MMP max N=212 |
| --- | --- | --- | --- | --- | --- | --- | --- | --- |
| Cortical adrenal insufficiency | 12 (2%) | 29 (14%) | 3 (1%) | 16 (2%) | 11 (9%) | 34 (10%) | 7 (2%) | 19 (9%) |
| Cataract | 4 (1%) | 19 (9%) | 6 (2%) | 17 (3%) | 7 (6%) | 28 (8%) | 6 (2%) | 20 (9%) |
| Diabetes | 11 (2%) | 32 (15%) | 7 (3%) | 15 (2%) | 14 (12%) | 39 (11%) | 7 (2%) | 26 (12%) |
| Hypertension | 27 (5%) | 38 (18%) | 7 (3%) | 25 (4%) | 19 (16%) | 63 (18%) | 9 (3%) | 42 (20%) |
| Obesity | 169 (37%) | 86 (42%) | 64 (29%) | 133 (21%) | 48 (41%) | 137 (39%) | 65 (19%) | 77 (37%) |
| Steroid-induced weight gain | 0(0%) | 112 (52%) | 0(0%) | 0(0%) | 84 (71%) | 201 (57%) | 0(0%) | 139 (66%) |
| Steroid-induced disorder of skin | 16 (3%) | 36 (17%) | 11 (5%) | 40 (6%) | 16 (13%) | 67 (19%) | 21 (6%) | 54 (25%) |
| Osteoporosis | 0(0%) | 145 (68%) | 0(0%) | 0(0%) | 58 (49%) | 215 (61%) | 0(0%) | 122 (58%) |

Abbreviations: OCS, oral corticosteroid,

MMP u – Unclustered multimorbidity, MMP ster – Steroid-associated multimorbidity, MMP all – Allergy-associated multimorbidity, MMP sn- Sinonasal-associated multimorbidity, MMP ster-all – Steroid+allergy-associated multimorbidity, MMP ster-sn – Steroid+sinonasal-associated multimorbidity, MMP all-sn – Allergy+sinonasal-associated multimorbidity, MP max – maximal multimorbidity


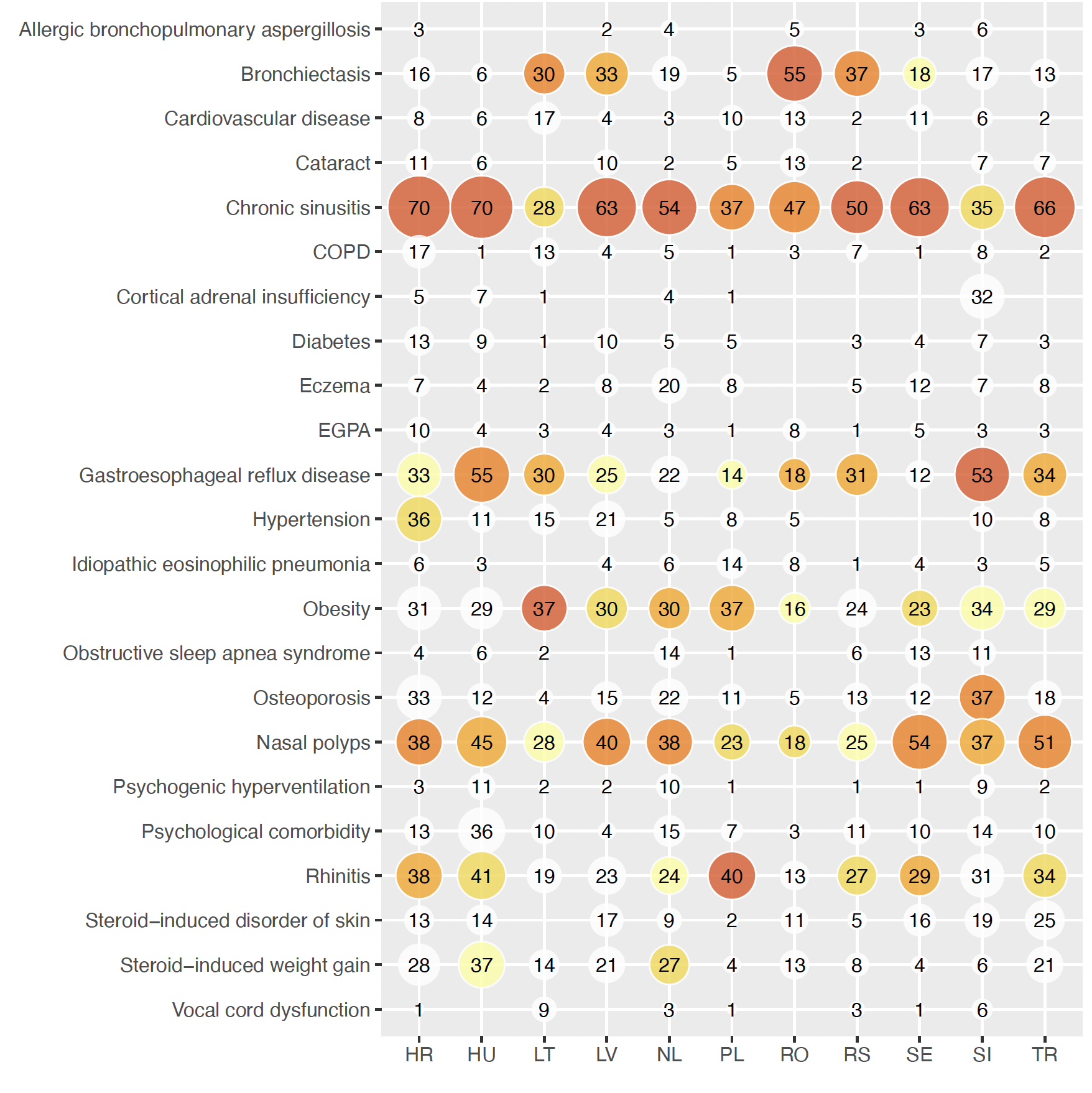


**Supplementary Figure 1: Percentage prevalence of comorbidities by country**

Abbreviations: COPD; Chronic Obstructive Pulmonary Disease, EGPA; eosinophilic granulomatosis with polyangiitis, HR; Croatia, HU; Hungary, LT Lithuania, LV; Latvia, NL; Netherlands, PL; Poland, RO; Romania, RS; Serbia, SE; Sweden, SI, Slovenia; TR, Türkiye; Size of bubble reflects percentage of whole, colours highlight top five comorbidities in each country - darker colour reflects higher prevalence.


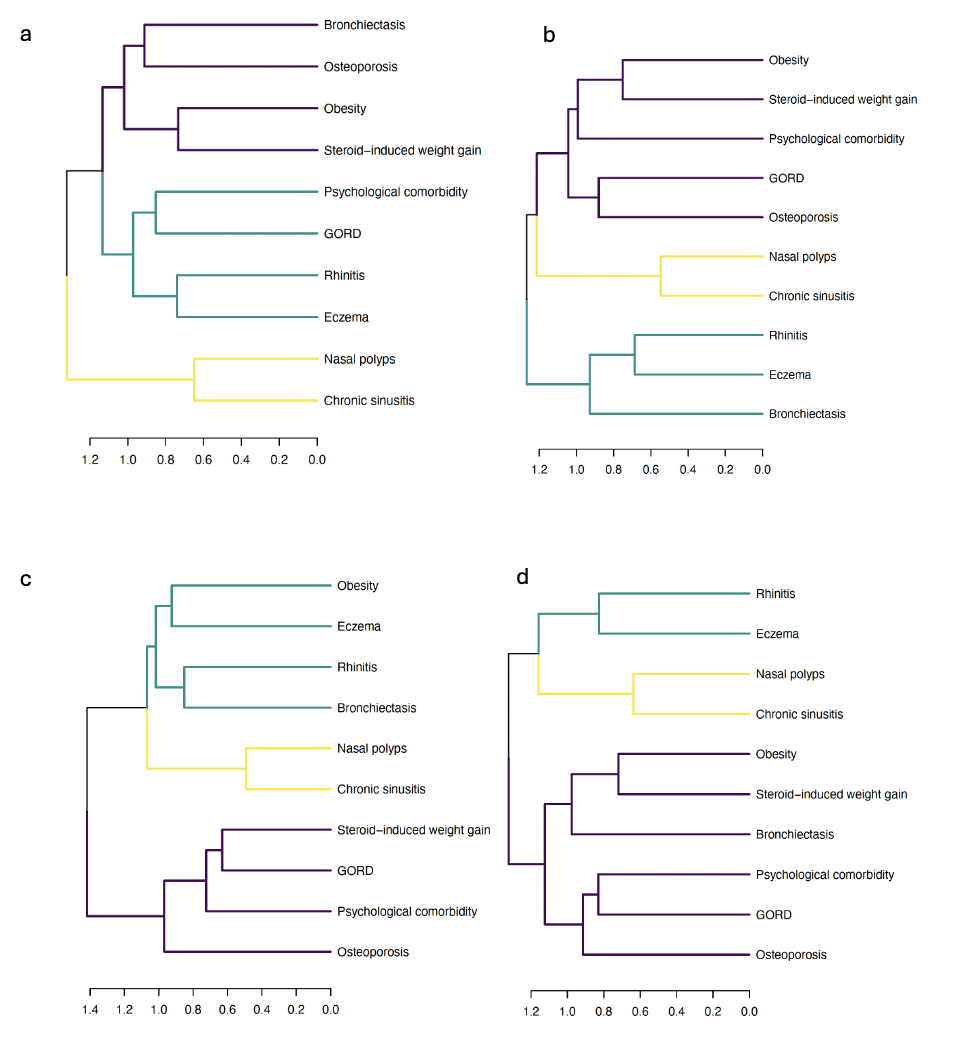


**Supplementary Figure 2: Dendrogram of comorbidity clustering across the four geographical regions (a) West, b) North, c) East, d) South)**

North (Lithuania (LT), Latvia (LV), Sweden (SE); East (Hungary (HU), Poland (PL) Romania (RO); South (Croatia (HR), Serbia (RS), Slovenia (SL), Türkiye (TR)); West (Netherlands (NL)).


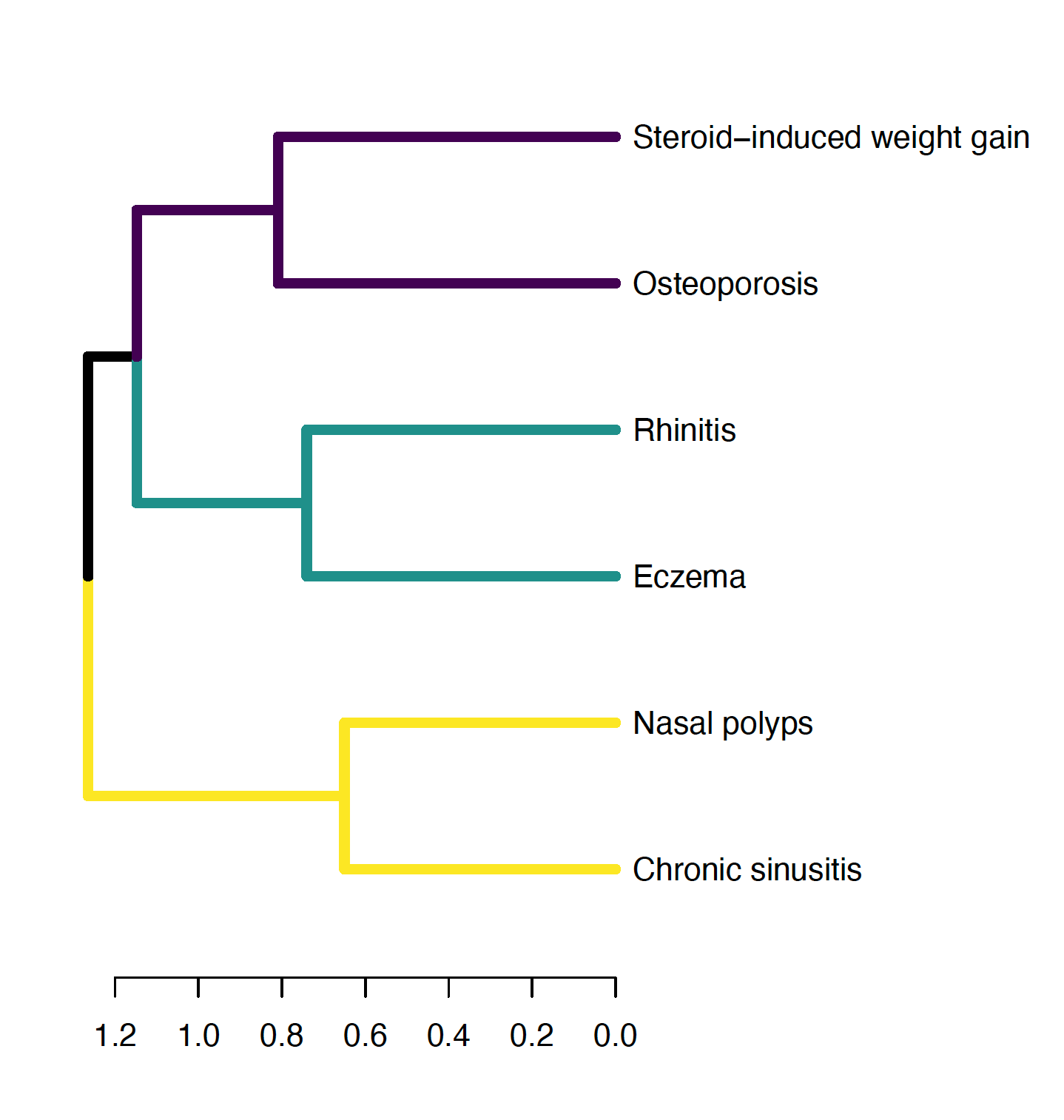


**Supplementary Figure 3: Dendrogram of consensus comorbidity clustering**


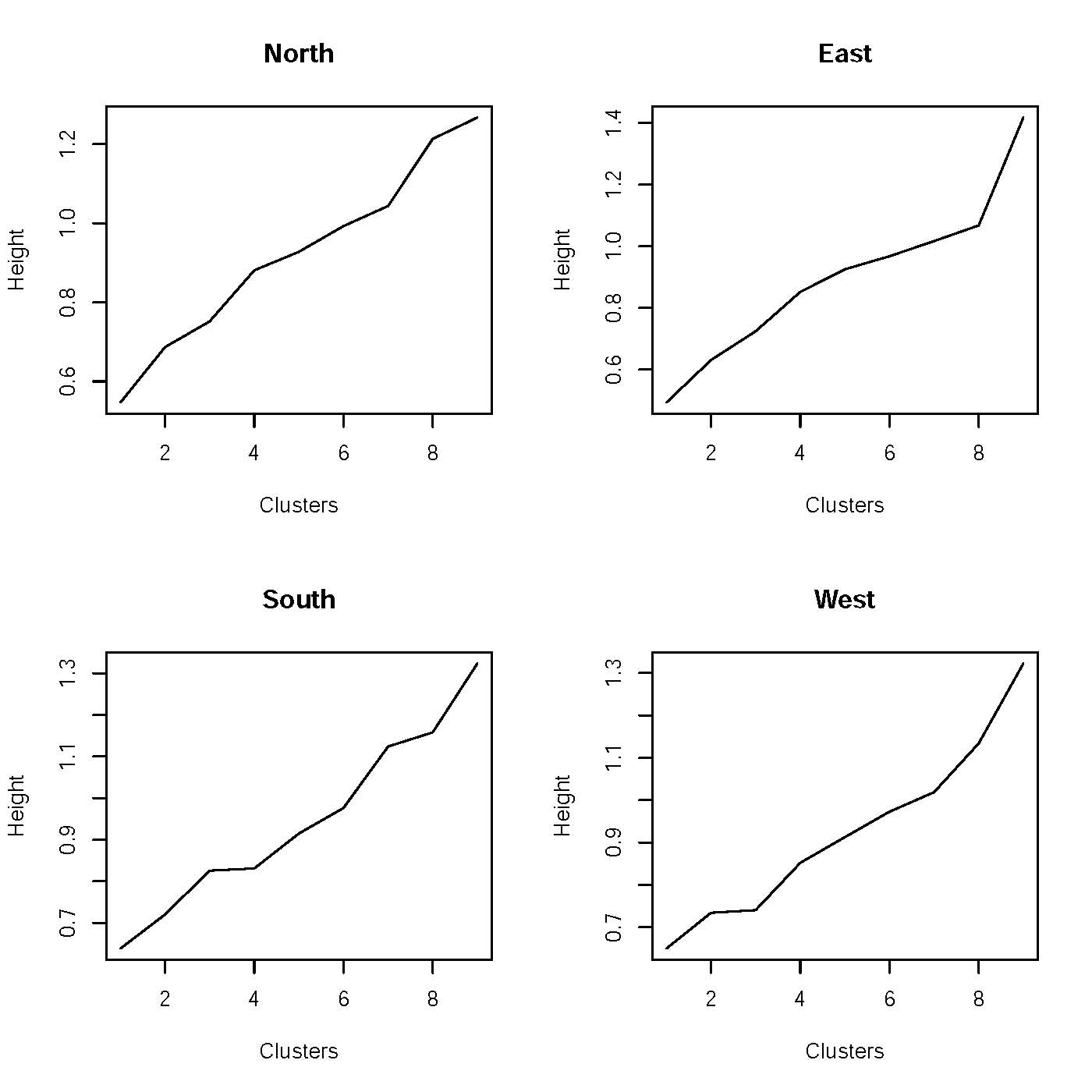


**Supplementary Figure 4: Scree plots, by region**

The Height (y-axis) represents the homogeneity of the cluster (aggregation criterion).  It is a correlation ratio, defined using the first principal component of the variables within that cluster.

**Supplementary Figure 5: Shows IIC for three factors when only anchor comorbidities are considered.**


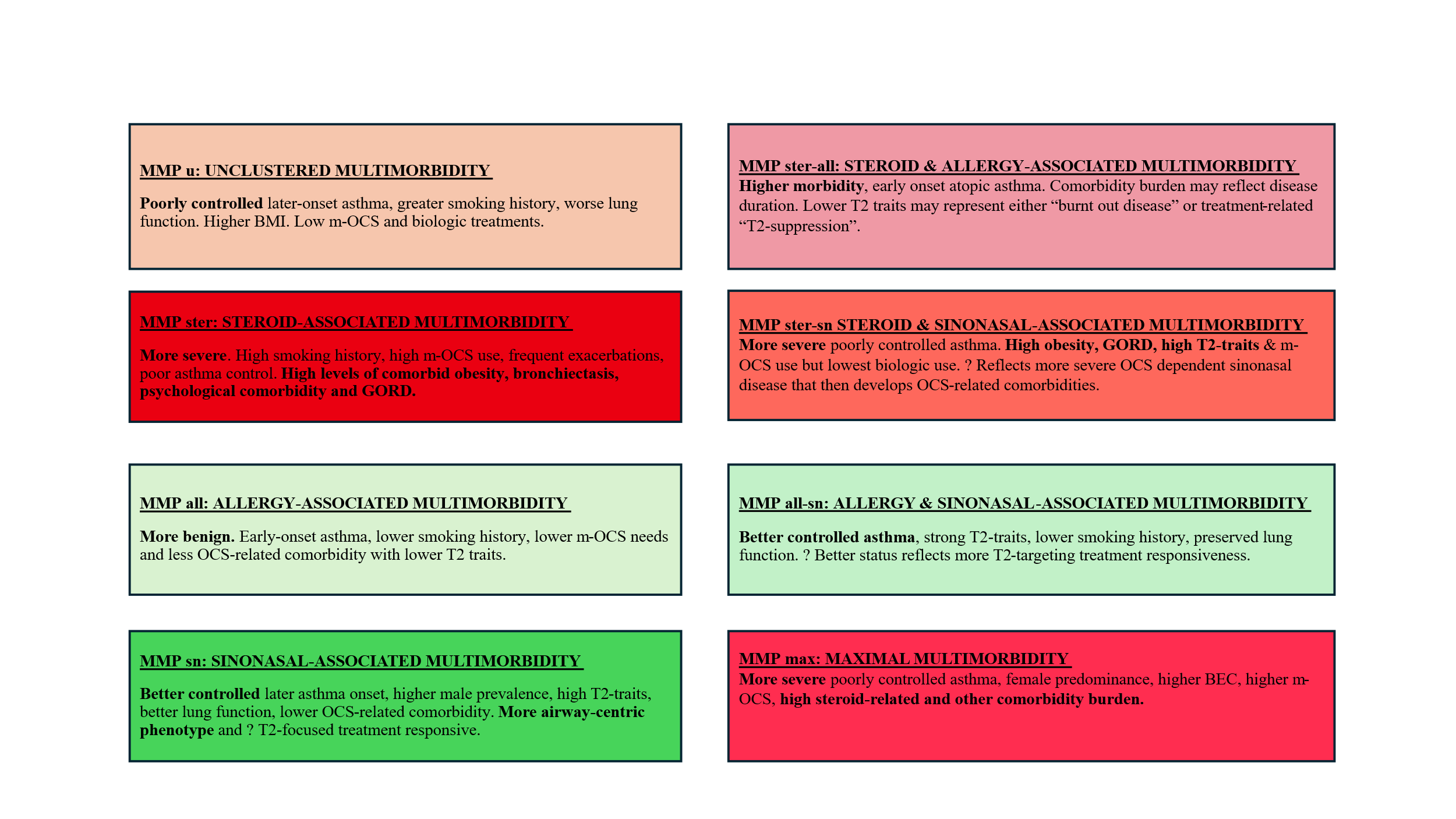


**Supplementary Figure 6: Clinical relevance of Multimorbidity Phenotypes**

Abbreviations: BEC, blood eosinophil count, GORD, gastroesophageal reflux disease, MMP, multimorbidity phenotypes, OCS, oral corticosteroids, T2, Type 2.
