# Supplementary material for: Multimorbidity phenotypes and associated characteristics in severe asthma: an observational study of European severe asthma registries
Source: Lancet Reg Health Eur. 2026 Feb 5;63:101600. doi: 10.1016/j.lanepe.2026.101600 (PMC12906202; doi:10.1016/j.lanepe.2026.101600)
Supplement: Supplymentary Material [file mmc2.pdf]

SHARP Registry

Severe Heterogenous Asthma Registry Patient-centered
- Central Registry -

Observational Study of the European Respiratory Society (ERS) Clinical Research Collaboration (CRC) SHARP

Study protocol

Version 1.0
30 August 2019

Sponsor:
SHARP CENTRAL foundation (NL)

Primary investigators:
SHARP chairs and steering Committee (appendix 2)

Table of Contents:

List of Abbreviations: 4
1. Overview/summary 5
  Type of Study 5
  Study period 5
  Study objectives 5
  Study population 5
  Study design 5
  Number of subjects 5
  Inclusion criteria 6
  Exclusion criteria 6
  Assessments and measures 6
2. Detailed study description 6
  Background 6
  Summary of Published Information 6
  Rationale 7
  Objectives 8
  Participant Selection 9
  Consent Procedures 9
  Registry Procedures 9
  Registry Duration 10
  Registry Infrastructure 10
  Materials of Human Origin: Collection, Preparation, Handling and Shipping 11
  Data Management and Quality Plan 11
  Sample Size Determination 12
  Registry Data Points 12
  Statistical Analysis Plan 12
  Potential Risks and Benefits 12
  Early Withdrawal of Subjects 13
  Adverse Event Reporting 13
  Ethical Considerations 14
  Sharing of Results with Subjects 14

Funding Source ... 14
Monitoring trial progress ... 14
Forms and data handling ... 14
Modification of the protocol ... 14
Administrative responsibilities ... 15
Publication Plan ... 15
References ... 15
APPENDIX 1: CRF ... 18
APPENDIX 2: primary investigators ... 33

List of Abbreviations:

ACQ	Asthma Control Questionnaire
AQLQ	Asthma related Quality of Life Questionnaire
CIS	Checklist individual strength
CRC	Clinical Research Collaboration
DPIA	Data Privacy Impact Analysis
eCRF	electronic Case Report Form
ERS	European Respiratory Society
HCI	Health Care Utilization
SAQ	Severe asthma questionnaire
SHARP	Severe Heterogeneous Asthma Registry Patient-centred
WPAI	Work Productivity and Activity Impairment
GCP	Good clinical practice

1. Overview/summary

Protocol Title:
SHARP CENTRAL registry

Type of Study
Prospective and retrospective real-life observational study

Steering committee:
See Appendix 2

Study centres:
Contributing study sites throughout Europe

Study period
Prospective, open-ended study. Study participants are asked at the time of signing up for SHARP CENTRAL Registry (= at the baseline visit) to attend as many annual follow up visits as possible

Study objectives
- To collect retrospective and prospective data on the phenotypical characteristics of patients with severe asthma, treatment strategies and patient responses to treatment, burden of severe asthma across Europe at baseline and during follow-up.
- To obtain natural history data on a wide spectrum of severe asthma patients
- To expedite identification and recruitment of participants for clinical trials
- To plan for future research studies (observational and interventional trials aimed at better symptom control and improving quality of life).

Study population
Study participants will be adults with diagnosis of severe asthma according to international guidelines. All study participants must be able to provide informed consent.

Study design
At the baseline study visit, patients will sign an informed consent form, and clinical data from the patient's medical files that are listed in appendix 1 will be collected and entered in the eCRF. Patients will also be asked to complete 6 questionnaires related to asthma.

At each annual follow up study visit, identical data to the ones at the baseline visit will be collected.

Number of subjects
This study will include as many eligible subjects as willing to participate. We hope to recruit at least 30 patients with severe asthma per 1,000,000 inhabitants residing in each individual country participating in the SHARP CENTRAL Registry within Europe within 6 years.

Inclusion criteria

All adults with a physician's diagnosis of severe asthma residing in one of the European countries are eligible to participate. Participants may be adult (18+) with no upper age limit, male or female. All participants must be able to provide informed consent for themselves.

Exclusion criteria

Subjects who are unable to understand the study protocol or unable to give informed consent.

Assessments and measures

See appendix 1

2. Detailed study description

Background

Asthma is a heterogeneous, inflammatory airway disorder with complex pathophysiological mechanisms (1). Although in most asthma patients the disease can be well controlled with inhaled anti-inflammatory and bronchodilator medications, this treatment fails in about 5-10% of patients (2). These patients develop severe asthma, which is a debilitating disease, associated with frequent severe exacerbations, emergency visits, and hospitalizations, resulting in a poor quality of life. For many years patients with this condition were dependent on the chronic use of high-dose inhaled and oral glucocorticoids, which put them at increased risk for serious and often life-threatening adverse events (3). However, recently, new biologics for severe asthma have become available (4). These biologics target key cytokines of so called "Type 2" airway inflammation, interleukin(IL)-4, IL-5 and IL-13. Mepolizumab and Reslizumab bind to interleukin (IL)-5, Benralizumab binds to the IL-5 receptor, while Dupilumab binds to the alpha subunit of the IL-4 receptor, thereby blocking IL4 and IL13 signalling. Treatments with these biologics have already resulted in remarkable improvements of asthma symptoms and quality of life, although, for reasons that are not understood, not everyone benefits despite meeting the approved inclusion criteria (4). However, many questions about the natural history, burden and costs of severe asthma, disease mechanisms, identification of different phenotypes, prediction of therapy response remain unanswered (5). These questions will be answered in studies organized by the ERS SHARP CRC, using data from SHARP CENTRAL Registry as well as other, already established, registries of patients with severe asthma in Europe.

Summary of Published Information

The past two decades have seen the creation of several European collaborations focusing on severe asthma, beginning in the late 90s with the Framework Programme (FP)-4 funded project on severe asthma, the European Network For Understanding Mechanisms Of Severe Asthma (ENFUMOSA), a cross-sectional observational study which provided the proof of principle for large collaborations (6). Soon after, a more in-depth project was established, focusing on mechanisms and biomarkers of severe asthma: Longitudinal Assessment of Clinical Course and BIOmarkers in Severe Chronic AIRway Disease (BIOAIR) project, funded by the FP-5 and FP-6

and several national funding bodies (7). More recently, the Unbiased BIOmarkers in PREDiction of respiratory disease outcomes (U-BIOPRED) programme, funded by the EU Innovative Medicines Initiative (IMI) project, sought to provide a step-change in phenotyping of chronic airways disease through the use of multi-dimensional clinical outcomes, ‘omics biomarkers and sophisticated lung imaging (8). The shared ambition of all these projects was to improve the definition of airways disease phenotypes in order to enable better targeting of existing drugs and those in development and to improve the understanding of determinants of disease severity, disease progression n and risk of exacerbations. These collaborations have produced more than 60 high impact publications in respiratory journals and numerous communications at international meetings. The concept of phenotype and endotypes, defined by shared clinical and underlying mechanistic features, respectively, is accepted both in respect of mechanisms and selection for treatment with biologics, for example anti-IL-5 antibody for eosinophilic forms of asthma (9).

During the same period, individual countries have also established national consortia, recognizing their essential role in bringing together the expertise that exists in individual centres and that this also brings people with asthma together. Some have focused on asthma and others have covered a spectrum of lung diseases: the Netherlands Severe Asthma Research Network, the British Thoracic Society Difficult Asthma Registry, the Belgian Severe Asthma Registry (10) the Spanish collaboration for asthma, the German Center for Lung Research, Poland’s Polastma (National Programme of Early Diagnosis and Therapy of Asthma (11)), the Finnish National Asthma Programme (12) and the United Kingdom’s National Institutes for Health Research (NIHR) Translational Research Collaborations in Inflammatory Respiratory Disease (TRC), all supported by various national public funding schemes. Several studies have shown that biomedical research which is carried out through international and interdisciplinary collaboration is of significantly higher quality and impact than research performed by a single centre or discipline. Thus, the national programmes in Finland, Poland and the UK (11,12,13) focusing on management and education, have resulted in marked improvements in clinical outcomes, showing the value of collaborative effort.

ERS SHARP CRC was conceived as a long-term project with the following mission (14): a) to gather essential information about the clinical and scientific expertise, existing capabilities and supporting infrastructure in centres across Europe, b) create a registry of people with severe asthma called SHARP CENTRAL registry and integrate it with other severe asthma registries in Europe, c) undertake research of relevance to people with severe asthma that can be best done through large collaborations.

ERS SHARP CRC builds on strong collaborative relationships among basic scientists, clinical investigators and patient organisations for severe asthma in the context of the European Respiratory Society. ERS SHARP CRC was reviewed and approved by the ERS Research Council, is sponsored by the ERS and receives additional funding from 5 pharmaceutical companies (GlaxoSmithKline, Sanofi, Chiesi, Novartis and TEVA.

Rationale

As severe refractory asthma is a relatively rare condition (15), no single study site is in the position to obtain clinical data in sufficient numbers to conduct conclusive studies concerning the majority of questions of clinical and patient relevance in real life severe asthma. Therefore, a cooperative effort throughout Europe appears an appealing avenue to provide large enough clinical data sets to answer questions conclusively by conducting sufficiently powered studies. Data from SHARP CENTRAL registry, being the central data registry of ERS SHARP CRC should assist in answering questions about (amongst others) the natural history of severe asthma, its burden and costs, underlying disease mechanisms, different phenotypes, prediction of therapy response, using independent real life data sets.

Objectives

Primary Objective

SHARP CENTRAL Registry is the central registry of ERS SHARP CRC and primarily designed to collect prospective data on the phenotypical characteristics of patients with severe asthma, treatment strategies and patient responses to treatment, burden of severe asthma across Europe at baseline and during follow-up.

Secondary Objectives

- To obtain natural history data on a wide spectrum of severe asthma patients
- To expedite identification and recruitment of participants for clinical trials
- To plan for future research studies (observational and interventional trials aimed at better symptom control and improving quality of life).

Research projects should aim to advance scientific knowledge towards optimizing treatment strategies that reduce the burden and costs of severe asthma, improve patients quality of life, and prevent poor outcomes.

The SHARP CENTRAL Registry database containing pseudo-anonymized data will be de-identified and integrated with other anonymized European severe asthma databases via a data integration platform controlled by the SHARP CRC. Data will be used for a variety of different analyses, which may be broadly categorized as either cross-sectional analyses or longitudinal analyses. SHARP CENTRAL Registry places no limit on the sample size to be collected or a timeframe in which the study will be completed. It is intended that data will be collected as long as needed to respond to pending questions defined by the SHARP CRC. The gradual amassing of phenotypical data will result in cumulative increases in statistical power in order to continuously improve the assessment tools that monitor the course of severe asthma and to identify clinical predictors of therapeutic responses, adverse events and patient relevant outcomes.

Participant Selection

Inclusion Criteria

SHARP CENTRAL Registry concerns adult patients (≥18 years) with severe asthma in the Europe. Severe asthma is defined according to international ERS/ATS guidelines (16). Briefly, this includes all adult asthma patients with uncontrolled symptoms for at least 6 months despite treatment with medium/high doses of inhaled corticosteroids, with or without oral corticosteroids, in combination with at least one other controller medication (e.g. long acting beta-2-agonists, theophylline), and having been checked for adherence to the medications, inhalation technique and co-morbid factors. These patients qualify for additional treatments for severe asthma, such as biologics. Some patients will already be using these therapies. Patients must be capable of giving informed consent and be able to travel to office/hospital for study visits.

Exclusion Criteria

Patients with alcohol/substance abuse within the past 6 months, or serious mental illness that might preclude subject's ability to comply with treatment will be excluded.

Consent Procedures

After speaking with a qualified nurse, physician or researcher who has provided an overview of the registry, if interested, the patient will be given a copy of the patient information sheet and the informed consent form to review. Study participants will be offered two levels of consent: a) to enter the registry for research purposes, and b) to allow future contact for invitation to participate in additional research studies. Additionally, patient contact information will be collected to facilitate future communication.

The patient will be informed that their participation in the registry is voluntary and will not in any way change their relationship with their own treating physician. Sufficient time (which may differ between European countries) for review of the patient information sheet and the informed consent paperwork will be given to allow the patient to request answers to any questions about the registry they may have. The patients will receive the contact information on who they can contact about these questions.

The informed consent form will then be signed by the patient or the legal representative and the person obtaining informed consent. The patient will be provided a copy of the signed and dated consent.

Registry Procedures

Specific Training

The research registry study team and all other persons assisting with the registry will be adequately trained about the protocol, the registry procedures, and their duties and functions.

Recruitment of participants

Patients contacting the asthma clinic to schedule an appointment for evaluation of their (severe) asthma will be informed about the registry and will be given the option to participate. When the patient comes to the clinic for their visit, information regarding registry participation will be provided by a qualified research staff member. This oral information will be provided while the patient is in a secluded area where confidentiality is guaranteed.

Data Collection Schedule

All data points collected for the registry will be collected as part of the asthma clinic. Investigators will be asked to evaluate participants at least once a year. The study calls for the documentation of annual assessments, with predefined range of tolerance of ± 1 month.

Enrolment

Prior to the initial visit, the patient will be consented. Initial baseline demographics of age, gender and ethnicity will be collected from clinic records. Medical history will be collected from clinic records and patient interview. Data from physical examination, diagnostic tests, and current medication use will be taken from clinic records. Patients will be asked to complete 6 questionnaires during clinic visits.

Subsequent yearly appointments: vital signs, height, weight, BMI, pulmonary function tests and current medications will be collected. The study subjects will be asked to complete 6 questionnaires either at the clinic, at home or digitally. All additional registry data (e.g. diagnostic tests) will be collected according to standard of care.

If and when the SHARP CRC develops additional questionnaires or diagnostic tests, these will be added but the patient will have the right to opt out of these and continue with only the original set of questionnaires and diagnostic tests.

Registry Duration

Patients will remain active in the registry until they voluntary withdraw or expire. Registry enrolment will be ongoing.

Registry Infrastructure

SHARP CENTRAL Registry will use an already existing electronic health record infrastructure designed for disease monitoring (CASTOR EDC). Castor EDC is a secure, cloud-based data solution, enabling researchers to easily capture and integrate data from any source. Thousands of medical device, biotech, and academic researchers around the world are using Castor EDC (Electronic Data Capture) to accelerate their studies. More information can be obtained from https://www.castoredc.com. Within SHARP CENTRAL Registry each participating country will have its separate databases. Institutes and user accounts for data-entry will be created within each country-specific database so that research workers have only access to records of their own site.

In addition, data can be directly obtained from patients using online questionnaires with e-health devices that can be connected with CASTOR EDC and are compliant with relevant regulations, such as GDPR and ISO 27001. An e-health system that is under development for that purpose is the PatientCoach system of the Leiden University Medical Center in The Netherlands.

Materials of Human Origin: Collection, Preparation, Handling and Shipping

The current projects conducted by the SHARP CRC do not include any collection of samples at individual sites for centralised analysis but if and when projects that do require additional samples, these will be handled according to standard good clinical practice (GCP) principles.

Data Management and Quality Plan

Data De-identification, Confidentiality, Storage, and Retention

Participant data are entered into the eCRF after creating a unique pseudonym for each participant. The pseudonym will consist of the first 3 letters of the country, 3 letters of the institute plus an automatically generated serial number. The identifying information will be stored in a secure place and be only accessible to the primary investigator of the individual participating centre. This person will only access the identifying information in case of medical urgency. The identifying information will never be stored electronically.

Registry documentation and paperwork will be stored at the asthma clinic in a locked file cabinet. Registry records will be retained as long as required by GCP rules in each country”
after the completion of the registry. After that period of time, all individual patient information will be shredded.

In order to make the data from SHARP CENTRAL Registry available for analysis the country-specific databases containing pseudo-anonymized data will be exported, anonymised and merged into a single datafile that can be used with standard statistical software packages. For integration with other anonymized European severe asthma databases a SHARP CENTRAL MySQL database, containing the merged tables, will be hosted at the Leiden University Medical Center. This MySQL database will allow connection with the data integration platform controlled by the SHARP CRC. This platform allows mapping of the SHARP CENTRAL Registry database to the OMOP (Observational Medical Outcomes Partnership) Common Data Model (CDM) which will form the basis for federated analyses based on SHARP CENTRAL Regsitry and other anonymized European severe asthma regsitries. OMOP is supported by the OHDSI collaborative framework (www.ohdsi.com). OHDSI provides resources to convert a wide variety of datasets into the CDM, as well as a plethora of tools to take advantage of the data once it is in CDM format. This includes Atlas a web based, open source software tool for researchers to conduct scientific analyses on standardized observational data.

Data Quality

The data entry forms in CastorEDC contain questions with coded answer categories (check boxes and radio-groups) where possible and input fields for quantitative data with plausible range checks and alerts. Quality control procedures for this research register will also include remote control visits. This means that source data will be verified by randomly selecting register participant

records, and comparing data from the original patient file with data in the electronic database record. If errors are common, data will be completely checked prior to data analysis.

Data Sharing

The study participant will give informed consent to his/her hospital and SHARP CENTRAL Foundation. The hospital will be responsible for obtaining the informed consent.

SHARP CENTRAL foundation and the hospital will be jointly responsible for the data. They will conclude a Data Sharing Agreement.

Sample Size Determination

SHARP CENTRAL registry places no limit on the overall sample size to be collected. However, each project that will be carried out with data from the SHARP CENTRAL Registry defining a specific-read out or endpoint will include a sample size calculation and – if appropriate – a power analysis specific to the objectives of the study.

Registry Data Points

Initial baseline demographics of age, gender and ethnicity will be collected from clinic records. Medical history (age of asthma onset, hospital admissions, ICU admissions, co-morbidities) will be collected from clinic records and patient interview.

Data from physical examination (height, length, blood tests, sputum tests, skin prick tests, pulmonary function test, Chest X-ray, Chest CT, DEXA scan, bronchoscopy, medication use will be taken from clinic records.

Questionnaires: Asthma control questionnaire (ACQ) (17), Severe asthma questionnaire (SAQ) (22).
Additional Questionnaires that are set up for the project “SAQ-Burden of Asthma” and that could potentially be added for future SHARP studies:
Asthma quality of life questionnaire (AQLQ) (18), Work productivity and activity impairment (WPAI) (19), Health care utilization (HCI) (20), Checklist individual strength (CIS) (21).
The questionnaires will be completed by the patient either at the clinic, at home or by a e-device at least once a year.

Statistical Analysis Plan

Overall, data from SHARP should assist in answering specific research questions, using independent data sets. As a result, each project proposal will include a detailed statistical analysis plan.

Potential Risks and Benefits

Potential Benefits

There are no direct benefits to study participants in this research registry. However, information regarding characteristics related to severe asthma will be obtained.

Potential Risks

This research represents a registry documenting severe asthma. The most likely risk posed to study participants would be a breach of confidentiality if someone other than the research team gains access to their data.

Mitigation of Risks

In each participating centre, there will be security measures in place to prevent breach of confidentiality from happening (e.g. locked cabinets, password protected files).

Provisions to Protect the Privacy Interest of Registry Participants

Precautions to protect the privacy of participants in SHARP will be taken according to the EU General Data protection Regulation (GDPR) summarized in a Data Privacy Impact Analysis (DPIA). All precautions will be taken to make sure that only authorized individuals will be accessing study participants research records. The collection of sensitive information about subjects is limited to the amount necessary to achieve the aims of the research registry, so that no unneeded sensitive information is being collected.

Early Withdrawal of Subjects

Investigator Withdrawal of Subjects

Patients are followed every year in the severe asthma clinic as part of their standard of care. If a patient does not return to the clinic for any regularly scheduled visit, the patient will not be withdrawn but visits will be considered as missing data in the data set. The investigator will have the right to withdraw individual patients if they have evidence that the patient is wilfully giving incorrect information about their asthma. Such withdrawals would have to be discussed and authorised by the national lead and – in case of a strong dispute – mediated by the SHARP CRC.

Research subject Request for Withdrawal from Registry

All study participants can withdraw from the study if they so choose. Patients wishing to withdraw from the research registry will be directed to contact the principal investigator in their study site. From the point of withdrawal, no further data will be collected from the patient or their medical records. However, any data collected up to the point of withdrawal will be maintained for integrity of the research registry. Patients will be made aware of their rights in the informed consent form.

Data Collection and Follow-up for Withdrawn Subjects

Patients who request withdrawal or who are withdrawn by the PI from the registry will have their data maintained in the research database up to the point of withdrawal. This data will be included in subsequent analysis.

Adverse Event Reporting

The primary purpose of the registry is NOT to systematically document adverse events from treatments, but to follow patients with severe asthma to understand more about the disease. However, since many study participants will be exposed to novel biological treatments for severe asthma, adverse events from these treatments may occur. Therefore, the investigator will seek

information on adverse events at each contact with the study participant, by specific questioning and, as appropriate, by examination. Information on all adverse events should be recorded in the source document and in the SHARP eCRF. All adverse events will be reported to local and national authorities as well as to European Medicines Agency (EMA) and the pharmaceutical industry that produces the biological.

Ethical Considerations

n/a.

Sharing of Results with Subjects

Patients will be regularly informed about the results of SHARP studies following practices in different countries.

Funding Source

SHARP CENTRAL Registry will be supported by funding from the European Respiratory Society (ERS). In the event that ERS funding ends, the participating centers will jointly attempt to obtain further funding from other sources in order to continue the registry.

Monitoring research progress

For data control there will be continuous evaluation of data for plausibility. There will be additional remote monitoring to check source documents and data entry. This means that original patient records will be compared with the electronic CRF. The investigator should allocate adequate time for these visits and should ensure that the monitor is given direct access to the patient source documents (e.g. hospital files). Between on-site monitoring visits the monitor should regularly check the electronic data for completeness and plausibility of the data. Missing data will be marked.

Forms and data handling

A complete CRF is attached in the Appendix. The data are entered electronically via internet-based technology. The SHARP CENTRAL Registry web-portal is separated into several parts with different access rules. Any given site investigator in SHARP CENTRAL Registry is allowed to see only data on study participants under the care of the study site to which the site investigator is affiliated. SHARP CRC is allowed to view all data of all centres for plausibility checks, quality control and monitoring. The whole database is saved on a server.

Modification of the protocol

Any modification of the protocol which may have an impact on the conduct of the study, including study objectives, study design, study participant population, study procedures or significant administrative aspects, will require a formal amendment to the protocol and approval by the ethics committee according to individual country rules.

Administrative responsibilities

The Investigator is responsible for the adequate medical care of the participant during the study. The Investigator must follow GCP Guidelines and is responsible for the safety and the medical care of the participant.

A contract will be issued to regulate the obligations and rights of the investigator and the responsibilities of SHARP CENTRAL foundation; the contract will be signed between authorized representatives of the respective institutions with which the investigators are affiliated and SHARP CENTRAL foundation, a non-profit charity registered in the Netherlands.

Access to the clinical database is regulated by the policies of SHARP CRC. Researchers interested in obtaining data for further analysis have to submit brief outlines of their severe asthma related research project to the Scientific Review Board of the SHARP CRC. The Scientific Review Board will assess whether the proposed project falls within the subject area to which study participants gave their informed consent and whether the proposal is ethically and scientifically sound. Once a project is approved by the Scientific Review Board, the proposer has to confirm in writing to comply with the data access and publication policy of SHARP and will provide a short abstract on the approved proposal for display at the SHARP web portal. Researchers conducting an approved project will then be granted to use a recoded excerpt of the clinical database for their analyses.

Publication Plan

The publication policy of SHARP is described in detail in a separate document: “SHARP Publication Process”

References

1: Papi A, Brightling C, Pedersen SE, Reddel HK. Asthma. Lancet. 2018;391(10122):783-800.

2: Israel E, Reddel HK. Severe and Difficult-to-Treat Asthma in Adults. N Engl J Med. 2017;377(10):965-976.

3: Sullivan PW, Ghushchyan VH, Globe G, Schatz M. Oral corticosteroid exposure and adverse effects in asthmatic patients. J Allergy Clin Immunol. 2018;141(1):110-116.

4: Bel EH, Ten Brinke A. New Anti-Eosinophil Drugs for Asthma and COPD: Targeting the Trait! Chest. 2017;152(6):1276-1282.

5: Pavord ID, Beasley R, Agusti A, Anderson GP, Bel E, Brusselle G, Cullinan P, Custovic A, Ducharme FM, Fahy JV, Frey U, Gibson P, Heaney LG, Holt PG, Humbert M, Lloyd CM, Marks G, Martinez FD, Sly PD, von Mutius E, Wenzel S, Zar HJ, Bush A. After asthma: redefining airways diseases. Lancet. 2018;391(10118):350-400.

6: Selroos O, Kupczyk M, Kuna P, Łacwik P, Bousquet J, Brennan D, Palkonen S, Contreras J, FitzGerald M, Hedlin G, Johnston SL, Louis R, Metcalf L, Walker S, Moreno-Galdó A, Papadopoulos NG, Rosado-Pinto J, Powell P, Haahtela T. National and regional asthma programmes in Europe. Eur Respir Rev. 2015;24(137):474-83.

7: Kupczyk M, Haque S, Sterk PJ, Niżankowska-Mogilnicka E, Papi A, Bel EH, Chanez P, Dahlén B, Gaga M, Gjomarkaj M, Howarth PH, Johnston SL, Joos GF, Kanniess F, Tzortzaki E, James A, Middelveld RJ, Dahlén SE; BIOAIR investigators. Detection of exacerbations in asthma based on electronic diary data: results from the 1-year prospective BIOAIR study. Thorax. 2013;68(7):611-8.

8: Wheelock CE, Goss VM, Balgoma D, Nicholas B, Brandsma J, Skipp PJ, Snowden S, Burg D, D'Amico A, Horvath I, Chaiboonchoe A, Ahmed H, Ballereau S, Rossios C, Chung KF, Montuschi P, Fowler SJ, Adcock IM, Postle AD, Dahlén SE, Rowe A, Sterk PJ, Auffray C, Djukanovic R; U-BIOPRED Study Group. Application of 'omics technologies to biomarker discovery in inflammatory lung diseases. Eur Respir J. 2013;42(3):802-25.

9: Eger KA, Bel EH. The emergence of new biologics for severe asthma. Curr Opin Pharmacol. 2019;46:108-115

10: Schleich F, Brusselle G, Louis R, Vandenplas O, Michils A, Pilette C, Peche R, Manise M, Joos G. Heterogeneity of phenotypes in severe asthmatics. The Belgian Severe Asthma Registry (BSAR). Respir Med. 2014;108(12):1723-32.

11: Kuna P, Kupczyk M, Kupryś-Lipińska I. POLASTMA--the Polish National Programme of Early Diagnosis and Therapy of Asthma. Pneumonol Alergol Pol. 2014;82(6):597-607.

12: Haahtela T, Herse F, Karjalainen J, Klaukka T, Linna M, Leskelä RL, Selroos O, Reissell E. The Finnish experience to save asthma costs by improving care in 1987-2013. J Allergy Clin Immunol. 2017;139(2):408-414.

13: Sweeney J, Brightling CE, Menzies-Gow A, Niven R, Patterson CC, Heaney LG; British Thoracic Society Difficult Asthma Network. Clinical management and outcome of refractory asthma in the UK from the British Thoracic Society Difficult Asthma Registry. Thorax. 2012;67(8):754-6.

14: Djukanovic R, Adcock IM, Anderson G, Bel EH, Canonica GW, Cao H, Chung KF, Davies DE, Genton C, Gibson-Latimer T, Hamerlijnck D, Heuvelin E, Louis R, Korn S, Kots M, Kwon N, Naddaf R, Wagers SS; SHARP Clinical Research Collaboration; Members of the CRC-SHARP. The Severe Heterogeneous Asthma Research collaboration, Patient-centred (SHARP) ERS Clinical Research Collaboration: a new dawn in asthma research. Eur Respir J. 2018;52(5).

15: Hekking PP, Wener RR, Amelink M, Zwinderman AH, Bouvy ML, Bel EH. The prevalence of severe refractory asthma. J Allergy Clin Immunol. 2015 Apr;135(4):896-902.

16: "International ERS/ATS guidelines on definition, evaluation and treatment of severe asthma." Kian Fan Chung, Sally E. Wenzel, Jan L. Brozek, Andrew Bush, Mario Castro, Peter J. Sterk, Ian M. Adcock, Eric D. Bateman, Elisabeth H. Bel, Eugene R. Bleecker, Louis-Philippe Boulet, Christopher Brightling, Pascal Chanez, Sven-Erik Dahlen, Ratko Djukanovic, Urs Frey, Mina Gaga, Peter Gibson, Qutayba Hamid, Nizar N. Jajour, Thais Mauad, Ronald L. Sorkness and W. Gerald Teague. Eur Respir J 2014; 43: 343-373. Eur Respir J. 2018;52(1).

17. Juniper EF, O'Byrne PM, Guyatt GH, Ferrie PJ, King DR. Development and validation of a questionnaire to measure asthma control. Eur Respir J. 1999;14(4):902-7

18; Juniper EF, Guyatt GH, Cox FM, Ferrie PJ, King DR. Development and validation of the Mini Asthma Quality of Life Questionnaire. Eur Respir J. 1999;14(1):32-8.

19; Chen H, Blanc PD, Hayden ML, Bleecker ER, Chawla A, Lee JH; TENOR Study Group. Assessing productivity loss and activity impairment in severe or difficult-to-treat asthma. Value Health. 2008;11(2):231-9.

20; Vollmer WM, Markson LE, O'Connor E, Sanocki LL, Fitterman L, Berger M, Buist AS. Association of asthma control with health care utilization and quality of life. Am J Respir Crit Care Med. 1999;160(5 Pt 1):1647-52.

21; Vercoulen JH, Swanink CM, Fennis JF, Galama JM, van der Meer JW, Bleijenberg G. Dimensional assessment of chronic fatigue syndrome. J Psychosom Res. 1994;38(5):383-92.

22: Hyland ME, Jones RC, Lanario JW, Masoli M. The construction and validation of the Severe Asthma Questionnaire. Eur Respir J. 2018;52(1). pii: 1800618.

APPENDIX 1: Case Report Form

[to be completed for every patient who is diagnosed with severe asthma]

Treating physician details
Name:
Hospital:

Has patient given informed consent for entering the registry
- ☐ Yes
- ☐ No

Date of entrance in registry (dd-mm-yyyy):

Is the patient currently participating in a (randomized) trial in which he/she receives study medication for asthma?
- ☐ Yes
- ☐ No

Date of randomization (dd-mm-yyyy):

Year/month of birth (yyyy/mm):

Gender
- ☐ Male
- ☐ Female

Ethnic background
- ☐ Caucasian
- ☐ Non-Caucasian
- ☐ Asian

Age at which doctor first diagnosed asthma _______ yr

[To be completed once every year by patient]

☐ Asthma control questionnaire (ACQ)[1]
Mean score _______

- ☐ Asthma Quality of Life Questionnaire (AQLQ)[2]
  Mean score _______

- ☐ Work productivity and Activity Impairment (WPAI)[3]
  Mean score _______

- ☐ Health care utilization Questionnaire[4]
  Mean score _______

- ☐ Checklist Individual Strength [5]
  Mean score _______

- ☐ Severe asthma Questionnaire [6]
  Mean score _______

[To be completed once every year by case-manager]

Is patient lost to follow-up?

- ☐ yes
  - ○ Patient passed away
  - ○ Patient changed email address
  - ○ Patient withdrew consent
  - ○ Other__________________
- ☐ No

Date of examination (dd-mm-yyyy):

Height: __________ cm
Weight: __________ Kg
BMI: __________ Kg/m2

Smoking Status:

- ☐ Never Smoker
- ☐ Ex-Smoker (Quit smoking >12 months)
- ☐ Current Smoker

Current number of Pack years: __________

Has patient ever been admitted to ICU for asthma?

- ☐ Yes
- ☐ No

If yes, total number of ICU admissions:

Has patient ever been intubated due to asthma?

- ☐ Yes
- ☐ No

If yes, total number of intubations:

Has the patient ever attended rehabilitation

- ☐ Yes
  - o How often? _____ times
  - o Last time _____ years ago
- ☐ No
- ☐ Unknown

Has the patient ever undergone bronchial thermoplasty?

- ☐ Yes

Date/year _____

- ☐ No
- ☐ Unknown

Has spirometry ever been performed (If done multiple times, please report most current result)

- ☐ Yes
- ☐ No

If Yes, when was most recent pulmonary function test performed? (dd/mm/yyyy)

If Yes, which values are available?

- ☐ Pre-bronchodilator values
- ☐ Post-bronchodilator values

	PRE BRONCHODILATOR		POST BRONCHODILATOR	
FEV1	L	% predicted	L	% predicted
FVC	L	% predicted	L	% predicted
FEV1/FVC	%		%	

Has exhaled NO testing ever been performed?

- ☐ Yes
- ☐ No

If yes, please report date of most recent result:
FeNO _______ppb

Has CO diffusion capacity been performed (ever)?

- ☐ Yes
- ☐ No

If yes, please report date of most recent result:

DLCO: _____ mmol/min/kPa and _______ % predicted

Has bodyplethysmography (body box) ever been performed?

- ☐ Yes
- ☐ No

If yes, please report date of most recent result:

TLC: _____ L and _______ % predicted
RV: _____ L and _______ % predicted

Has bronchial provocation testing with methacholine ever been performed ?

- ☐ Yes
- ☐ No

If yes, please report date of most recent result:

- ○ Positive (BHR demonstrated, FEV1 decline ≥20%)
- ○ Negative (BHR not demonstrated, FEV1 decline <20%)

Has blood cell differential counting been performed in the last 5 years?

- ☐ Yes
- ☐ No

If yes, please report date of most recent result:
- Leukocytes: ____ x 10E9 and ____%
- Neutrophils: ____ x 10E9 and ____%
- Eosinophils: ____ x 10E9 and ____ %

If yes, what was the highest eosinophil count ever measured?

<u>Has blood glucose or HbA1c been measured during the last year?</u>

☐ Yes
☐ No

If yes, please report date of most recent result:
Fasting glucose level:
Hba1c level

<u>Has Blood Gas Analysis been performed</u>/

☐ Yes
☐ No

If yes, please report date of most recent result:
pH ______ pCO2: ______ kPa / ______ mmHg; pO2: ______ kPa / ______ mmHg;

<u>Has total serum IgE testing been performed?</u>

☐ Yes
☐ No

If yes, please report date of most recent result:
______ IU/ml or kU/l

<u>Has a routine allergy test RAST/CAP or skin prick testing even been performed?</u>

☐ Yes, positive
☐ Yes, negative
☐ No

If yes, please report date of most recent result:

Has allergy to aspergillus ever been tested?

- ☐ Yes, positive
- ☐ Yes, negative
- ☐ No

If yes, please report date of most recent result:

Has allergy to food ever been tested?

- ☐ Yes, positive
- ☐ Yes, negative
- ☐ No

If yes, please report date of most recent result:

Has sputum analysis (cell counts) ever been performed?

- ☐ Yes
- ☐ Yes, but inadequate sample
- ☐ No

If yes, please report date of most recent result:
_______ % Eosinophils
_______ % Neutrophils

Has sputum culture been performed during last year?

- ☐ Yes, positive for pathogens
- ☐ Yes, negative for pathogens
- ☐ Yes, throat bacteria
- ☐ No

If yes, please report date of most recent result:

Has Chest X-Ray ever been performed?

- ☐ Yes, findings compatible with asthma
- ☐ Yes, findings compatible with other pulmonary disorders
- ☐ Yes, normal findings
- ☐ No

If yes, please report date of most recent result:

Has Chest HRCT ever been performed

- ☐ Yes

- ☐ No

If yes, please report date of most recent result:

- ☐ Bronchiectasis
- ☐ Emphysema
- ☐ Tree-in-bud configuations
- ☐ Other pulmonary abnormailties
- ☐ No abnormal findings

Has DEXA-scan ever been performed

- ☐ Yes, pathological findings
- ☐ Yes, normal findings
- ☐ No

If yes, please report date of most recent result:

Has bronchoscopy ever been performed

- ☐ Yes, findings compatible with asthma
- ☐ Yes, findings compatible with other lung diseases
- ☐ Yes, normal findings
- ☐ No

If yes, please report date of most recent result:

Does the patient have one of the following co-morbidities

Atopic dermatitis

- ☐ Yes
- ☐ No or very unlikely
- ☐ Only in the past
- ☐ Unknown

Allergic rhinoconjunctivitis

- ☐ Yes
- ☐ No or very unlikely
- ☐ Only in the past
- ☐ Unknown

Chronic rhinosinusitis

- ☐ Yes
- ☐ No or very unlikely
- ☐ Only in the past
- ☐ Unknown

Nasal polyposis

- ☐ Yes
- ☐ No or very unlikely
- ☐ Unknown

Aspirin intolerance

- ☐ Yes
- ☐ No or very unlikely
- ☐ Unknown

Vocal cord dysfunction

- ☐ Yes
- ☐ No or very unlikely
- ☐ Only in the past
- ☐ Unknown

Hyperventilation syndrome and/or panic disorder

- ☐ Yes
- ☐ No or very unlikely
- ☐ Only in the past
- ☐ Unknown

Depression

- ☐ Yes
- ☐ No or very unlikely
- ☐ Only in the past
- ☐ Unknown

Gastroesophageal reflux

- ☐ Yes
- ☐ No or very unlikely
- ☐ Only in the past
- ☐ Unknown

Frequent (>2/yr) respiratory infections requiring antibiotics?

- ☐ Yes
- ☐ No or very unlikely
- ☐ Only in the past
- ☐ Unknown

Bronchiectasis

- ☐ Yes
- ☐ No or very unlikely
- ☐ Unknown

EGPA (Churg-Strauß-Syndrome)

- ☐ Yes
- ☐ No or very unlikely
- ☐ Unknown

Eosinophilic pneumonia

- ☐ Yes
- ☐ No or very unlikely
- ☐ Only in the past
- ☐ Unknown

ABPA (Allergic bronchopulmonary aspergillosis)

- ☐ Yes
- ☐ No or very unlikely
- ☐ Unknown

Chronic heart failure

- ☐ Yes
- ☐ No or very unlikely
- ☐ Only in the past
- ☐ Unknown

OSA (Obstructive sleep Apnea)

- ☐ Yes
- ☐ No or very unlikely
- ☐ Only in the past
- ☐ Unknown

Other co-morbidities

Has adherence to therapy ever been checked?

- ☐ Yes
- ☐ No

If yes, please report date of most recent result:

% ICS taken (per period checked): _______%

Has Inhalation technique been checked?

- ☐ Yes
- ☐ No

If yes, date last instruction:

Does the patients currently take one or more of the following medications

If yes, which one and at what dose?

☐ SABA
- o salbutamol ≤ 8 puffs/day
- o salbutamol > 8 puffs/day
- o terbutaline ≤ 8 puffs/day
- o terbutaline > 8 puffs/day

☐ Anticholinergics
- o ipratropium

☐ Fixed SABA/anticholinergic combinations
- o ipratropium / fenoterol ≤ 8 puffs/day
- o ipratropium / fenoterol > 8 puffs/day
- o ipratropium / salbutamol ≤ 8 puffs/day
- o ipratropium / salbutamol > 8 puffs/day

☐ LABA single inhaler
- o formoterol_____ug/day
- o salmeterol_____ug/day
- o indacaterol_____ug/day
- o olodaterol_____ug/day

☐ ICS single inhaler
- beclomethasone _____ug/day
- budesonide_____ug/day
- fluticasone_____ug/day
- ciclesonide _____ug/day

☐ LAMA single inhaler:
- tiotropium: _____ µg/day
- glycopyrronium:_____ µg/day
- aclidinium: _____ µg/Day

☐ Fixed ICS/LABA combination inhaler
- budesonide / formoterol
  budesonide ____ µg/day andfFormoterol____ µg/day
- salmeterol / fluticasone
  salmeterol ____ µg/day and Fluticasone propionate____ µg/day
- beclomethasone / formoterol
  beclomethasone ____ µg/day and formoterol ____ µg/day
- fluticasone / formoterol
  fluticasone ____ µg/day and formoterol ____ µg/day
- fluticasone furoate/ vilanterol
  fluticasone fuorate ____ µg/day and vilanterol ____ µg/day

☐ Fixed LABA/LAMA combination inhaler
- indacaterol / glycopyronium
  indacaterol ____ µg/day and glycopyronium____ µg/day

☐ montelukast

☐ theophylline

☐ Maintenance oral corticosteroids
- predniso(lo)ne_____ mg/day
- dexamethasone _____ mg/day
- betamethasone _____ mg/Day
- hydrocortisone _____ mg/Day
- triamcinolone (injections) _____ mg/month

Is patient currently tapering his/her oral/systemic corticosteroid dose?

☐ Yes
☐ No

<u>Is the patient currently being treated with biologic therapy?</u>

☐ Yes, omalizumab
Start date: dd/mm/yyy

Dose per injection visit_____ mg

Frequency of dosing_____ weeks

Has omalizumab dose or interval been changed in last 12 months:
☐ Yes, because of adverse effects
☐ Yes, because of insufficient response
☐ Yes, because of (very) good response
☐ No

☐ Yes, mepolizumab
Start date: dd/mm/yyy

Dose per injection/visit_____ mg

Frequency of dosing_____ weeks

Has mepolizumab dose or interval been changed in last 12 months:
☐ Yes, because of adverse effects
☐ Yes, because of insufficient response
☐ Yes, because of (very) good response
☐ No

☐ Yes, reslizumab
Start date: dd/mm/yyy

Dose per infusion/visit_____ mg

Frequency of dosing_____ weeks

Has reslizumab dose or interval been changed in last 12 months:
☐ Yes, because of adverse effects
☐ Yes, because of insufficient response
☐ Yes, because of (very) good response

- ☐ Yes, dupilumab
  Start date: dd/mm/yyy

  Dose per injection/visit_____ mg

  Frequency of dosing_____ weeks

  Has dupilumab dose or interval been changed in last 12 months:
  - ☐ Yes, because of adverse effects
  - ☐ Yes, because of insufficient response
  - ☐ Yes, because of (very) good response
  - ☐ No

- ☐ Yes, benralizumab
  Start date: dd/mm/yyy

  Dose per injection/visit_____ mg

  Frequency of dosing_____ weeks

  Has benralizumab dose or interval been changed in last 12 months:
  - ☐ Yes, because of adverse effects
  - ☐ Yes, because of insufficient response
  - ☐ Yes, because of (very) good response

- ☐ No, patient is currently not treated with biologics

<u>Does the patient currently use any of the following (non-pulmonary) medications?</u>

- ☐ Nasal corticosteroids
- ☐ Antihistamines
- ☐ Bisphosphanates
- ☐ Calcium suppletion (± vit D)
- ☐ Proton pump inhibitors
- ☐ vit K antagonists
- ☐ NSAIDs
- ☐ Other

<u>Has the patient received one of the following asthma add-on treatments IN THE PAST?</u>

☐ omalizumab
If Yes, date of last administration (dd/mm/yyyy)
If yes, reason for stopping?
- ☐ insufficient reduction of systemic steroid dose
- ☐ Adverse effects
- ☐ Insuffcient improvement of symptoms or exacerbations
- ☐ Insufficient improvement of lung function

☐ mepolizumab
If Yes, date of last administration (dd/mm/yyyy)
If yes, reason for stopping?
- ☐ insufficient reduction of systemic steroid dose
- ☐ Adverse effects
- ☐ Insuffcient improvement of symptoms or exacerbations
- ☐ Insufficient improvement of lung function

☐ reslizumab
If Yes, date of last administration (dd/mm/yyyy)
If yes, reason for stopping?
- ☐ insufficient reduction of systemic steroid dose
- ☐ Adverse effects
- ☐ Insuffcient improvement of symptoms or exacerbations
- ☐ Insufficient improvement of lung function

☐ benralizumab
If Yes, date of last administration (dd/mm/yyyy)
If yes, reason for stopping?
- ☐ insufficient reduction of systemic steroid dose
- ☐ Adverse effects
- ☐ Insuffcient improvement of symptoms or exacerbations
- ☐ Insufficient improvement of lung function

☐ dupilumab
If Yes, date of last administration (dd/mm/yyyy)
If yes, reason for stopping?
- ☐ insufficient reduction of systemic steroid dose
- ☐ Adverse effects
- ☐ Insuffcient improvement of symptoms or exacerbations

    - ☐ Insufficient improvement of lung function
- ☐ methotrexate
- ☐ cyclosporine
- ☐ azathioprine
- ☐ cyclophosphamide
- ☐ interferon alpha
- ☐ aspirin desensitization
- ☐ macrolide therapy (>6 weeks)
- ☐ immunotherapy
- ☐ other: ________________________

Which steroid-induced adverse effects were ever objectively diagnosed?

- ☐ Skin bruising / thinning
- ☐ Weight gain
- ☐ Cataract
- ☐ Hypertension
- ☐ Osteoporosis
- ☐ Diabetes mellitus
- ☐ Depression
- ☐ Other: _______________________
- ☐ Other: _______________________
- ☐ Other: _______________________
- ☐ Other: _______________________

APPENDIX 2: primary investigators

Chairs

- Ratko Djukanovic. NIHR Southampton Centre for Biomedical Research, Southamptom, UK
- Elisabeth Bel. AmsterdamUMC, University of Amsterdam, Amsterdam, The Netherlands
- Dominique Hamerlijnck, European Lung Foundation, Sheffield, UK, Asthma UK, London, UK. Dutch Lung Foundation, Amersfoort, The Netherlands, EUPATI, Brussels, Belgium.
- Toni Gibson; European Lung Foundation, Sheffield, UK, Asthma UK, London, UK.

Steering Committee

- George Walter Canonica. Humanitas Humanitas Clinical and Research Center, Humanitas University, Rozzano, Milan, Italy
- Mina Gaga. Athens Chest Hospital Sotiria, Athens, Greece
- Liam Heaney. Queens University Belfast, Belfast, UK
- Ildiko Horvath. National Institute of Pulmonology, Budapest, Hungary
- Marc Humbert, Université Paris-Sud, Le Kremlin-Bicêtre, France
- Jan-Willem Kocks Unioversity of Groningen, Groningen,The Netherlands
- Stepahnie Korn. Mainz University Hospital, Mainz, Germany
- Piotr Kuna. Medical University of Lodz, Poland
- Renaud Louis, Liege Univesity, Liege Belgium
- David Ramos Barbon. Hospital Sant Pau &Biomedical research Institute Stant Pau, Barcelona, Spain
- Thomas Sandstrøm. Umeå University, Umeå, Sweden
